# Regulation of *HbPIP2;3*, a Latex-Abundant Water Transporter, Is Associated with Latex Dilution and Yield in the Rubber Tree (*Hevea brasiliensis* Muell. Arg.)

**DOI:** 10.1371/journal.pone.0125595

**Published:** 2015-04-30

**Authors:** Feng An, Zhi Zou, Xiuqing Cai, Jin Wang, James Rookes, Weifu Lin, David Cahill, Lingxue Kong

**Affiliations:** 1 Danzhou Investigation & Experiment Station of Tropical Crops, Ministry of Agriculture, Rubber Research Institute, Chinese Academy of Tropical Agricultural Sciences, Danzhou, 571737, P. R. China; 2 Institute for Frontier Materials, Deakin University, Geelong, 3216, Australia; 3 College of Agronomy, Hainan University, Haikou, 570228, P. R. China; 4 School of Life and Environmental Sciences, Deakin University, Geelong, 3216, Australia; Beijing Forestry University, CHINA

## Abstract

Rubber tree (*Hevea brasiliensis*) latex, the source of natural rubber, is synthesised in the cytoplasm of laticifers. Efficient water inflow into laticifers is crucial for latex flow and production since it is the determinant of the total solid content of latex and its fluidity after tapping. As the mature laticifer vessel rings are devoid of plasmodesmata, water exchange between laticifers and surrounding cells is believed to be governed by plasma membrane intrinsic proteins (PIPs). To identify the most important PIP aquaporin in the water balance of laticifers, the transcriptional profiles of ten-latex-expressed PIPs were analysed. One of the most abundant transcripts, designated *HbPIP2;3*, was characterised in this study. When tested in *Xenopus laevis* oocytes HbPIP2;3 showed a high efficiency in increasing plasmalemma water conductance. Expression analysis indicated that the *HbPIP2;3* gene was preferentially expressed in latex, and the transcripts were up-regulated by both wounding and exogenously applied Ethrel (a commonly-used ethylene releaser). Although regular tapping up-regulated the expression of *HbPIP2;3* during the first few tappings of the virginal rubber trees, the transcriptional kinetics of *HbPIP2;3* to Ethrel stimulation in the regularly tapped tree exhibited a similar pattern to that of the previously reported *HbPIP2;1* in the virginal rubber trees. Furthermore, the mRNA level of *HbPIP2;3* was associated with clonal yield potential and the Ethrel stimulation response. Together, these results have revealed the central regulatory role of *HbPIP2;3* in laticifer water balance and ethylene stimulation of latex production in *Hevea*.

## Introduction

Natural rubber, an indispensable industrial raw material for producing high performance rubber products, is formed from the latex harvested through tapping the perennial tropical rubber tree (*Hevea brasiliensis* Muell. Arg.)[1]. The latex represents the cytoplasmic content of laticifers which are organized as concentric mantels inside the secondary phloem of the tree trunk. Water accounts for 60%–70% of the latex upon each tapping [2]. Sufficient water supply is essential for both latex regeneration and latex flow [3, 4]. Therefore, any conditions favouring water supply of a rubber tree, especially water movement into phloem, could contribute to latex flow and production. The transmembrane water movement into cells was previously explained only by simple diffusion [5]. However, since it could not explain many phenomena, such as the high velocity of water exchange, the remarkable variations between different cell types and the transient change of water permeability subjected to the stimulation of some reagents [5], the existence of hydrophilic pores within biological membranes was proposed [6]. Following the identification and characterisation of the channel forming integral protein (CHIP28), an aquaporin from human erythrocytes [7, 8], our knowledge on molecular basis of transmembrane water flow has significantly increased [9]. Subsequently, numerous aquaporin genes have been cloned and functionally confirmed to play pivotal roles in plant transmembrane water transport [9, 10] and whole plant water balance [11–14].

Aquaporins are ubiquitous water channel proteins embedded in intracellular and plasma membranes that regulate transmembrane water flow [15]. Aquaporins belong to the major intrinsic proteins (MIP) superfamily, and generally have an average molecular weight of 26 to 34 KDa [9, 13]. Based on sequence similarities and subcellular localizations, plant aquaporins were previously divided into four subfamilies, i.e., plasma membrane intrinsic proteins (PIPs), tonoplast intrinsic proteins (TIPs), nodulin26-like intrinsic proteins (NIPs), and small basic intrinsic proteins (SIPs). Recently, three more subfamilies of plant aquaporins, *viz*. GlpF-like intrinsic proteins (GIPs), hybrid intrinsic proteins (HIPs), and uncategorized X intrinsic proteins (XIPs), have also been identified from the moss *Physcomitrella patens* [5, 16]. Although aquaporins were first identified as selective water channels, increasing evidence has demonstrated that they could also facilitate the permeability of some neutral molecules (for example: glycerol, urea, formamide and hydrogen peroxide), metalloids (e.g., silicon, boron, arsenite and antimony), and gases (e.g., ammonia, nitric oxide and carbon dioxide) [14, 17, 18]. Therefore, aquaporins can now be considered as multifunctional channels involved in various plant physiological processes, such as plant cell osmoregulation, leaf physiology, seed germination, root water uptake, plant reproduction and plant development [9, 14, 19–21].

In rubber trees, the mature laticifer vessel rings are devoid of functional plasmodesmata connections [22]. Therefore, the water circulation of laticifers, which is of major significance to latex flow and latex regeneration, relies largely on the transmembrane water channel activity mediated by aquaporins, especially plasma membrane-targeted PIPs [23]. According to sequence similarities and structural features, PIPs can be further divided into PIP1 and PIP2 subgroups [24]. In comparison to PIP1s, PIP2s generally have a shorter N-terminal tail and a longer C-terminal extension which usually harbors several additional phosphorylation sites [25]. When heterologously expressed in *Xenopus laevis* oocytes or yeast cells, PIP2s usually have high water channel activity, whereas PIP1s are inactive or are associated with relatively low water permeability [9, 23, 26–28]. Thus, PIP2s expressed in laticifer cells are thought to contribute the most to the water balance of the rubber tree laticifer system.

Although it has been reported that a high latex water content is normally linked to a high rubber tree yield potential [2, 29, 30] and that Ethrel stimulation may significantly dilute latex [2, 23], the molecular mechanism of water influx into laticifers and subsequent latex dilution following tapping and stimulation was unclear prior to the discovery of aquaporins in rubber trees. Based on a bark cDNA library, Tungngoen et al. [23, 28] isolated three full-length aquaporin cDNAs (denoted *HbPIP2;1*, *HbPIP1;1*, *HbTIP1;1*) and confirmed their involvement in hormone-induced latex dilution and yield promotion by using virginal (untapped) trees of an ethylene susceptible clone PB217 in which latex yield was up-regulated by ethylene stimulation. Meanwhile, our group cloned and characterized another two aquaporin genes (*HbPIP1* and *HbPIP2*) from rubber tree latex [31]. However, the characterization of rubber tree aquaporins is still in its infancy with much to be uncovered.

Rubber tree clones respond differently to Ethrel stimulation [23, 32, 33]. For example, clone PR107, which harbours a high level of sucrose content and TSC in the latex, responds well to Ether stimulation [34], whereas clone CATAS8-79 displays a relatively poor response [35]. In comparison to PR107 and CATAS8-79, CATAS7-33-79 illustrates a moderate comportments of TSC, sucrose content and response to Ethrel stimulation [29]. In addition, the TSC of rubber tree latex is normally linked to rubber tree yield potential [2, 29, 30] and Ethel stimulation response [32, 33]. Rubber tree clones with a low TSC usually exhibit high rubber yield potential but limited response to Ethrel. For instance, clone PB217 is a relatively late maturing variety which has a high TSC, short latex flow duration and low latex metabolism and responds very well to Ethrel stimulation as characterized by significantly prolonged latex flow, latex yield and high latex metabolism [23, 33]. Whereas, clones PB235 and PB260 are fast growing varieties which have low TSC and long latex flow duration, and do not respond much to Ethrel stimulation [33]. Therefore, the latex TSC difference among clones is probably linked to the expression of aquaporin genes. However, clonal variation in aquaporin expression has not been reported. Moreover, successive tappings at a regular interval result in a progressive increase in rubber tree latex yield but accompanied by a gradual decrease in latex total solid content (TSC) until an equilibrium after six to ten tappings is attained [1, 33]. The decrease in latex TSC with the first few tappings implies tapping influences the expression of aquaporins which raises the question whether Ethrel stimulation of regularly tapped trees has similar effect to that which has been reported on virginal rubber trees [23]. After all, Ethrel stimulation is only practically used several years after a rubber tree has been tapped [1, 33].

To identify the most important PIP aquaporins in the water flux of laticifers, the transcriptional profile of ten latex-expressed PIP transcripts obtained was surveyed based on the latex transcriptome data (SRA accession number SRX278514). Among the three most highly abundant transcripts, the one designated *HbPIP2;3* was novel and thus investigated in detail in this study. The high efficiency of water transport activity of *HbPIP2;3* was verified in *X*. *laevis* oocytes, and its expression profile upon tapping, wounding and Ethrel stimulation was analysed. Furthermore, the transcriptional kinetics of *HbPIP2;3* following Ethrel stimulation was studied by using two rubber tree clones with varying Ethrel-response. Importantly, our data indicates a central regulatory role of *HbPIP2;3* in laticifer water balance and Ethylene stimulation of latex production in *H*. *brasiliensis*.

## Materials and Methods

### Plant materials

The rubber trees were cultivated at the experimental farm of the Chinese Academy of Tropical Agricultural Sciences (Danzhou, Hainan, China). Clones of PR107, CATAS8-79 and CATAS7-33-97 were used as plant materials according to their distinct responses to Ethrel stimulation and plant material accessibilities. PR107 and CATAS8-79 clones were selected to study the *HbPIP2;3* expression kinetics subjected to Ethrel stimulation as they respond to Ethrel treatment differently [29, 34, 35]. All trees were budded rubber trees on the unselected rootstocks. These trees were planted in 2002 and had been regularly tapped for 3 years under the tapping system of s/2 d3 (tapping every 3 days with half spiral) without any stimulation before our experiments were performed.

CATAS7-33-97 rubber clone was used for the experiments related to virginal (untapped) trees, due to the unavailability of the virginal PR107 and CATAS8-79 clones. To study the effects of tapping and wounding on the expression of *HbPIP2;3*, eight-year-old virginal CATAS7-33-97 rubber trees were selected on their similarity in girth and growth performance similarity. They were opened to tap under the tapping system of s/2d/3. To investigate the tissue specific expression of *HbPIP2;3*, ten tissues, i.e. latex, bark, root, bud, leaves and their petioles of different developmental stages (e.g., bronze, mature and senescent), were collected from the eight-month-old tissue cultured polybag saplings produced from the secondary somatic embryogenesis derived CATAS7-3-97 rubber clone [36].

### Ethrel treatment and sampling

The kinetics of the Ethrel effect on latex parameters and aquaporin expressions were performed as described by Tungngoen et al. [23]. A total of 18 regularly tapped but not Ethrel stimulated trees were selected based on their similarity in girth, latex yield and dry rubber content. Then, the trees were randomly divided into six groups with each group contained 3 trees. One group was set up as the control (without Ethrel stimulation) and the other five groups were subjected to Ethrel treatments at different time point before sampling. The control group was subjected to 1 g of carboxyl methyl cellulose (CMC, 1%) with a brush above the tapping cut 24 h before tapping, while the five treatment groups were treated with 1g of 2.5% (w/w) Ethrel in 1% CMC in the same way as the control at a designated time (3 h, 6 h, 12 h, 24 h, 40 h) before tapping.

All the trees were tapped at 6:30 am on the same day. The tapping cuts were firstly cleaned carefully with tissues and the tapping knife was sterilized with 95% ethanol. The trees were then tapped and the latex within the first 45 min was dropped into liquid nitrogen after discarding the first 5 drops, whereas the bark slices were pooled and immediately frozen with the liquid nitrogen. All samples were quickly transported to the laboratory and stored at -70°C for total RNA isolation.

### Wounding, tapping, and Ethrel treatment on virginal trees

The effects of wounding, tapping, and Ethrel stimulations on *HbPIP2;3* expressions were examined on CATAS7-33-97 rubber trees as described by Tang et al. [37]. Briefly, 18 mature virginal trees were chosen for their similar girth (50.4 ± 0.9cm) and were divided into 3 groups. Each group was subjected to 1 g of 1% CMC (Control), 1 g of 2.5% Ethrel in 1% CMC (ET), or nine stainless steel drawing pins stuck into the bark at 3 cm intervals (DP), respectively. The treatments were performed 24 h before the first tapping, just above the planned tapping cut. All trees were tapped in a pattern of s/2d/3 in the early morning on the same days. Latex and bark samples were collected 6 tappings after the treatments.

### Latex yield and total solid content (TSC)

Latex yield was determined by measuring the volume of harvested latex after exudation had ceased. After latex yield determination, the latex was thoroughly mixed and 2 mL of the fresh latex sample was transferred into a pre-weighed glass bottle. The bottle and latex was then weighed and the latex dried at 75°C in a ventilated oven for at least 48 h to determine the dry matter content. The TSC was calculated as the ratio of dry and fresh matter expressed as a percentage [23].

### RNA isolation and cDNA synthesis

Latex, bark and different tissue samples were collected from a pool of three trees per treatment, and the total RNAs were isolated as described by Tang et al. [37]. After verifying the RNA purity and concentration with a spectrophotometer (DU-70, Beckman, USA), cDNA templates were synthesized by reverse transcribing the isolated RNAs with PrimeScript RT reagent kit (Takara, Dalian, China) according to the manufacturer’s protocol, and stored at -20°C until needed.

### Expressional analysis based on Solexa sequencing

Previously, the latex transcriptome was sequenced on the Solexa platform. Gene annotation and alignments indicated that 10 unigenes were putative aquaporin encoding sequences (see S1 File) in the 54495 unigenes longer than 200 bp obtained by SOAP *de novo* assembly [38]. Among the ten PIPs, *HbPIP1;1* (GenBank accession number GQ903902), *HbPIP2;1* (FJ851079), *HbPIP1;4* (GQ479823, previously denoted as *HbPIP1*) and *HbPIP2;7* (GQ479824, previously denoted as *HbPIP2*) were reported elsewhere [23, 28, 31]. Unigenes annotated as the plasma membrane intrinsic protein or PIP were used as queries to search for the genome of clone RRIM600 [39] using BLASTn [40]. The transcribed region of each PIP gene was defined with reads. If more than one unigenes were located in one locus, the full-length gene corrected with reads were used for further analysis. To analyse the expression profile of putative PIP genes, the latex transcriptome data (SRA accession number SRX278514) of RRIM928 [41] was downloaded from NCBI (http://www.ncbi.nlm.nih.gov/sra). The raw reads were firstly filtered by removing the adaptor reads, adaptor-only reads, low quality reads (reads containing more than 50% bases with Q-value≤5) and ambiguous sequences (reads with “N” rate larger than 10%). Then, the trimmed reads were mapped to the unigenes using Bowtie2 [42], and the RPKM (Reads per kb per million reads) method was used for expression annotation [43]. The formula is shown below:

$$RPKM=\frac{10^{9}C}{NL}$$ (1)

where RPKM (A) is set to be the expression of a certain unigene A; C is the number of reads that uniquely aligned to unigene A, N is total number of reads that uniquely aligned to all unigenes, and L is the number of bases on unigene A.

### Cloning and functional characterization of HbPIP2;3 in *Xenopus* oocytes

According to the *in silico* identified full-length cDNA, the coding region of *HbPIP2;3* was confirmed using the primer pair of 5'- GTA CAG ATC TAT GGT GAA GGA CGT GAC AGA AC -3' (forward) and 5'- GTA CAG ATC TTT AAA CAG TTG GGT TGC TCC TG -3' (reverse), where underlined nucleotides specify *Bgl*II restriction site.

The water channel activity of *HbPIP2;3* was determined using *X*. *laevis* oocytes [23]. Briefly, the ORFs of *HbPIP2;3* and *HbPIP2;1* (a functional proven aquaporin used as the positive control) were subcloned into the blunt-ended *Bgl*II site of the pXβG-ev1 expression vector (a gift from Dr. Tomoaki Horie). After confirming the insertions via both PCR and sequencing, the constructed plasmids were linearized with *Bam*HI restriction enzyme and the cRNA with cap analog [m7G(5′)ppp(5′)G] were synthesized *in vitro* using a mMESSAGE mMACHINE T3 High Yield Capped RNA Transcription Kit (Ambion, USA).

*Xenopus* oocytes were prepared, defolliculated, and stored in Barth’s solution [44]. To measure the water permeability coefficient (*P*_f_), 50 nL cRNA solution containing 50 ng each of *HbPIP2;3* and *HbPIP2;1* capped RNA or 50 nL of RNAse-free water (negative control) were firstly injected into the stage V and VI oocytes using a Pneumatic PicoPump (PV820, WPI). Subsequently, the injected oocytes were incubated at 20°C in modified Barth’ s solution (MBS, containing NaCl 88 mM, KCl 1 mM, CaCl_2_ 0.41 mM, Ca(NO_3_)_2_ 0.33 mM, MgSO_4_ 0.83 mM, NaHCO_3_ 2.4 mM, HEPES 10 mM, Penicillin 10 μg/mL, streptomycin 10 μg/mL, pH 7.4) for one day. The healthy oocyte individuals were then transferred from MBS (200 mOsm, Osm_in_) to the 1/5 MBS (40 mOsm, Osm_out_) for water influx (swelling) measurement. Oocyte diameter changes were recorded with an inverted microscope system (SZX16, Olympus). The *P*_*f*_ values were determined using the equation:

$$P_{f}=\frac{V_{0}}{S\times V_{w}\times \left({\text{osm}}_{in}-{\text{osm}}_{out}\right)}\frac{d\left(\frac{V}{V_{0}}\right)}{dt}$$ (2)

where *V*_*0*_ is the initial oocyte volume, *S* is the initial cell surface area, *V*_*w*_ is the molar volume of water (18 cm^3^∙mol^-1^), *osm*_*in*_ and *osm*_*out*_ are the internal and external osmolarities, respectively[23]. For the HgCl_2_ inhibition lines, 0.3 mM HgCl_2_ were added to the incubating MBS for 10 min before water permeability was measured. The swelling experiments were carried out from two independent experiments with each assayed on a minimum of 10 oocytes.

### Quantitative real-time PCR

Real-time PCR was performed using the *HbYLS8* gene as the housekeeping gene as it has been reported to be one of the most stably expressed reference genes in response to tapping, hormone treatment and other experimental conditions [45]. The gene specific primers were 5'-CGT TGG ATT GGG TGC CGA GAT-3' (forward) and 5'-CCA GGG CTT GTC CTG ATT GTA-3' (reverse) for *HbPIP2;3* and 5'- GGG CTC TCA AGG ACA AGC AA-3' (forward), 5'-GGA GCA ATA ACC AAA CCA CGA-3' (reverse) for *HbYLS8*. A SYBR-green Mix (Takara, Dalian, China) fluorescent dye and a 96 pore Real-time Thermal Cycler (Type 5100, Thermal Fisher Scientific Oy, Finland) were utilised for PCR reactions. The reaction program was: 30 s at 95°C for denaturation, followed by 40 cycles of 5 s at 95°C, 30 s at 60°C for amplification and then 30 s at 60°C for extension. Each 10 μL reaction mix contained 1μL template, 5μL 5×SYBR Premix, 0.3 μL (0.15 μL each) primers and 3.7 μL RNase-water. All assays were performed in triplicate. The amplification efficiency for each primer pair was estimated to be 0.99 and 1.01, respectively, and the amplified fragments were confirmed via Sanger sequencing. The relative abundance of aquaporins transcripts was estimated as 2^ΔΔt^ by PikoReal2.0 software unless otherwise specified.

### Statistical analysis

Statistical analyses were executed using the Data Processing System software v11.0. The differences among means were tested following Duncan’s one-way ANOVA (*P*<0.05).

## Results

### Cloning and sequence analysis of *HbPIP2;3*

**Fig 1:**
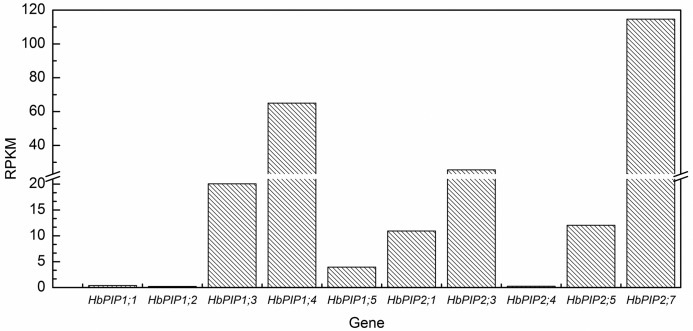
The transcriptional profile of ten PIP genes identified in the rubber tree latex. The expression analysis was based on the Solexa sequencing data of RRIM928 latex transcriptome downloaded from NCBI (SRA accession number SRX278514). RPKM refers to reads per kilobase per million (see details in methods). *HbPIP1;1* and *HbPIP2;1* were previously reported by Tungngoen et al. [23, 28], whereas *HbPIP1;4* and *HbPIP2;7* were reported as *HbPIP1* and *HbPIP2* by Zhuang et al. [31], respectively.

In order to identify the transcripts expressed in laticifers and determine the most important PIPs in water balance of laticifers, the latex (representing the laticifer cytoplasm) transcriptome of rubber tree clone RRIM928 was analysed. After cleaning and quality checking, about 47.7 million clean reads with an average length of 93 nt were retained from more than 50.3 million raw reads. Read-mapping supported the expression of ten-latex-expressed PIP genes identified before (see S1 File). The expression of *HbPIP1;1* and *HbPIP2;1* was very low, whereas *HbPIP2;7*, *HbPIP1;4* and *HbPIP2;3* were fairly abundant (Fig 1). Since PIP2s are shown to have high water channel activity and PIP1s are inactive or have low water permeability [9, 23, 26–28], the highly abundant *HbPIP2;3* and *HbPIP2;7* are proposed to play a more important role in the laticifer water balance. To obtain the full-length cDNA and investigate the gene structure of *HbPIP2;3*, the transcriptome and genome sequences were aligned. Results suggested that the transcriptional region of this gene is 2377 bp, containing three introns, and the cDNA is 1576 bp which harbors an 858 bp open reading frame (ORF). Then, the ORF was cloned and confirmed using gene specific primers. It is found that even though there are two SNPs (single nucleotide polymorphisms) between the isolated ORF and corresponding region of RRIM600 genome [39], the deduced protein is the same (see S2 File). The cDNA sequence was therefore submitted to NCBI GenBank under the accession number of KF921089.

**Fig 2:**
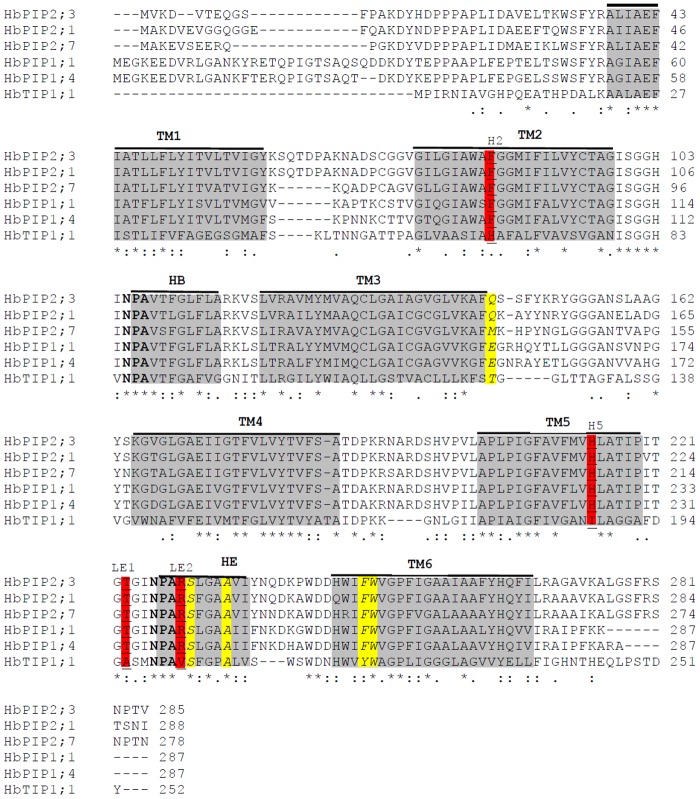
Multiple sequence alignment of HbPIP2;3 with aquaporins reported in rubber tree. Sequences were aligned using ClustalX2 [47]. Predicted transmembrane helices (TM1-TM6) and the two short helices forming NPAs (HB and HE) are shaded and labeled with black lines above the alignment. The two conserved NPA motifs are shown in bold letters. Residues comprising the ar/R filter are underlined and labeled H2, H5, LE1 and LE2. P1–P5 residues from N- to C-terminus are indicated by italic.

**Fig 3:**
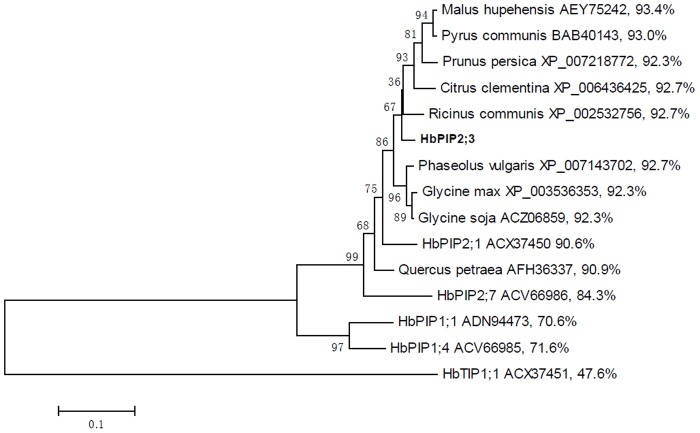
Phylogenetic analysis of HbPIP2;3 with other aquaporins. Predicted amino acid sequences were aligned using ClustalX2 [47] and the phylogenetic tree was constructed using bootstrap neighbour-joining tree (1000 replicates) method and MEGA4 software [48]. The distance scale denotes the number of amino acid substitutions per site. The similarity of each protein with HbPIP2;3 is shown on the right.

Sequence analysis indicated that *HbPIP2;3* putatively encoded 285 amino acids with a theoretical molecular weight (Mw) of 30.34 kDa and isolectric point (pI) of 9.18. According to multiple alignment, the deduced polypeptide of *HbPIP2;3* was shown to harbor several key characteristics of MIP family proteins, including six transmembrane helices (TM1–TM6) connected by five loops (LA–LE), and two highly conserved NPA motifs which are located at the N-terminal end of two half helices (HB and HE) formed by LB and LE, respectively. In addition, HbPIP2;3 harbors the ar/R selective filter of F-H-T-R, and five residues (Q-S-A-F-W) at Froger’s positions which represent a typical feature of water channel proteins as seen in HbPIP2;1 and other water transport activity proven aquaporins [23, 46] (Fig 2). Homology analysis revealed that HbPIP2;3 shares a similarity of more than 90% with its homologs in *Malus hupehensis* (AEY75242), *Pyrus communis* (BAB40143), *Ricinus communis* (XP_002532756), *Phaseolus vulgaris* (XP_007143702), *Citrus clementina* (XP_006436425), *Glycine max* (XP_003536353), *Prunus persica* (XP_007218772), *Glycine soja* (ACZ06859) and *Quercus petraea* (AFH36337). When compared with reported rubber tree aquaporins, HbPIP2;3 shares the highest similarity of 90.6% with HbPIP2;1 (ACX37450) and the lowest of 47.6% with HbTIP1;1 (ACX37451), respectively (Fig 3).

### Functional validation of HbPIP2;3

**Fig 4:**
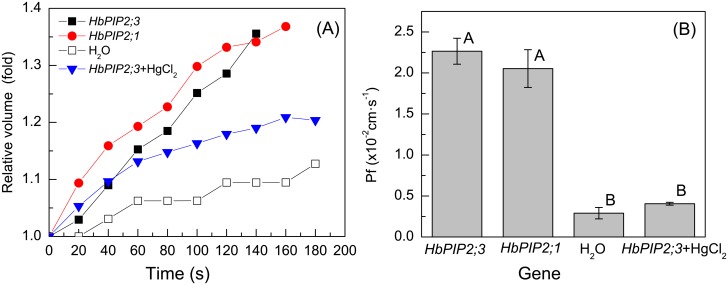
Water channel activity of HbPIP2;3. (A) Time-dependent cell volume changes and, (B) water permeability (P_f_) values of *Xenopus* oocytes expressing rubber tree aquaporins. The oocytes were injected with 50 ng *HbPIP2;3* and *HbPIP2;1* (positive control) cRNA solution or RNase-free water (negative controls) were incubated in full strength MBS for 24 h and then transferred into a 5-fold diluted MBS at time 0 for cell volume measurements. The cell volume change was recorded every 20 s for up to 200 s or until the cell burst. The oocytes for the HgCl_2_ inhibition group were subjected to treatment with MBS containing 0.3 mM HgCl_2_ 10 min before the swelling assay. The Pf data are given as mean ± SD (n = 10) from two independent experiments. Bars with different letters are significantly different (Duncan one-way ANOVA, *P*<0.05).

The high presence of *HbPIP2;3 and HbPIP2;7* prompted us to check their water channel activities. 50 ng of *HbPIP2;3*, *HbPIP2;7*, *HbPIP2;1* (positive control) cRNA solution or 50 nL of RNase-free water (negative control) were injected into *Xenopus* oocytes and their cell volume change monitored following transfer from a hypertonic solution into hypotonic solution. To our surprise, HbPIP2;7, which reported elsewhere, exhibited very low water channel activity when heterologously expression in *Xenopus* oocytes [49], even though it shares a high similarity of 85.1% with HbPIP2;1. Nevertheless, the time-dependent increase in cell volume (Fig 4A) shows that the cell volume of oocytes expressing *HbPIP2;3* increased as quickly as that of the functionally validated *HbPIP2;1* [23]. The calculated P_f_ values (Fig 4B) of the oocytes expressing *HbPIP2;3* and *HbPIP2;1* were similar, both being more than 5-fold greater than the oocytes injected with RNase free water. The water permeability of *HbPIP2;3* was inhibited by 0.3 mM HgCl_2_ as observed for most other plant aquaporins [50–53]. These results suggest that *HbPIP2;3* encode an efficient water channel sensitive to mercury indicating its importance in the water balance of rubber tree laticifers. Our further studies were, therefore, focused on *HbPIP2;3*.

### Tissue specificity of *HbPIP2;3*

**Fig 5:**
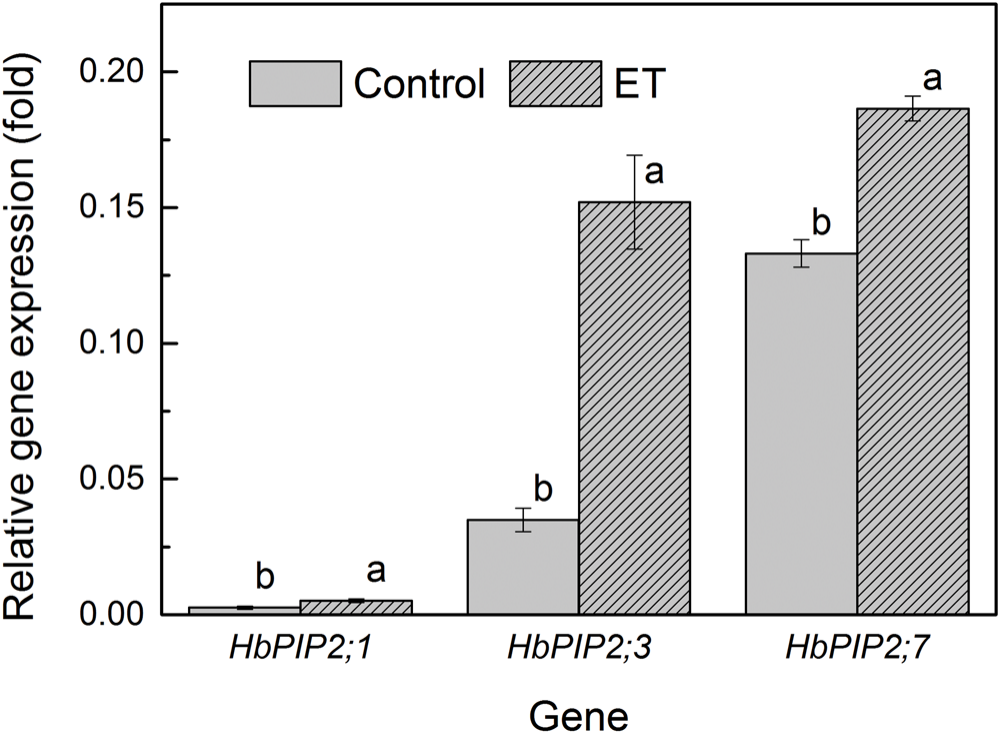
Relative transcript abundance of *HbPIP2;1*, *HbPIP2;3* and *HbPIP2;7* aquaporins in rubber tree latex. The relative gene expression is the ratio of each aquaporin transcript to the *HbYLS8* house-keeping gene using 2^Δt^ method. The PR107 rubber trees regularly tapped for three years without Ethrel stimulation were used as the plant materials. The Control rubber trees were brushed with only 1g of 1% CMC, while the 24h rubber trees were brushed with 1g of 2.5% (w/w) Ethrel in 1% CMC, at just above the tapping cut 24 hour before sampling. Different letters indicate a significant difference (*P*<0.05) in gene transcript expression between control and Ethrel-treated trees.

**Fig 6:**
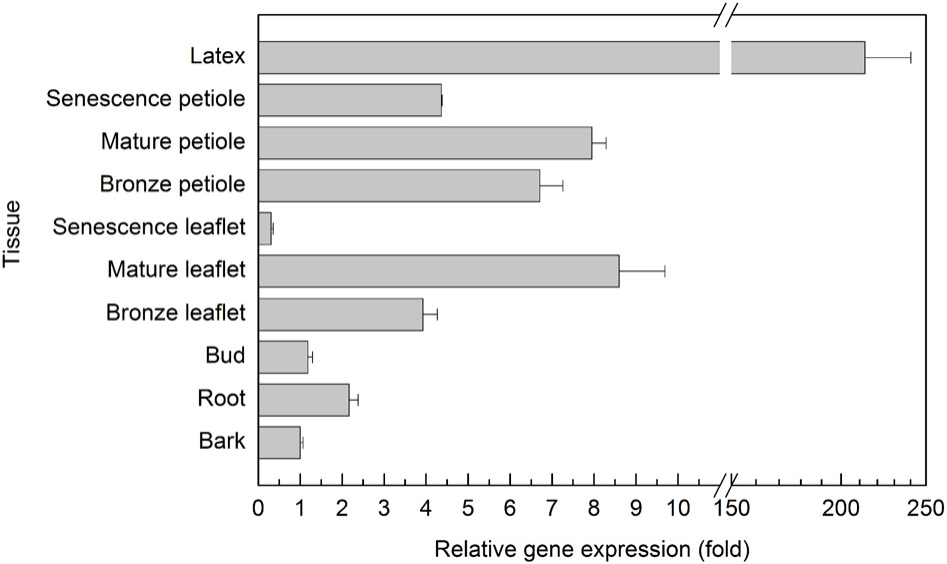
Expression of *HbPIP2;3* in different rubber tree tissues. The relative gene expression was calculated by using *HbYLS8* as the house-keeping gene and bark as the normalization sample. The tissues were obtained from tissue cultured CATAS7-33-97 rubber tree saplings (about eight-month-old). All quantitative RT-PCR assays were conducted in triplicate.

The expression of *HbPIP2;1*, *HbPIP2;3* and *HbPIP2;7* were compared with a quantitative RT-PCR in the PR107 rubber tree latex using the gene specific primers (S1 Table). It is confirmed in Fig 5 that *HbPIP2;3*, and *HbPIP2;7* were expressed to markedly higher levels in latex and can be regulated by Ethrel when compared to the previously reported *HbPIP2;1* [23]. Further, the expression of *HbPIP2;3* in ten different stage tissues was determined in CATAS7-33-97 rubber tree saplings. It can be observed from Fig 6 that *HbPIP2;3* was identified in all the examined organs and showed the highest presence in latex, which was more than 200-fold that of bark, suggesting its preferential expression in latex and a major role in laticifer water balance.

### Effect of wounding, tapping and Ethrel stimulation on *HbPIP2;3* expression and its association with latex dilution and yield

**Fig 7:**
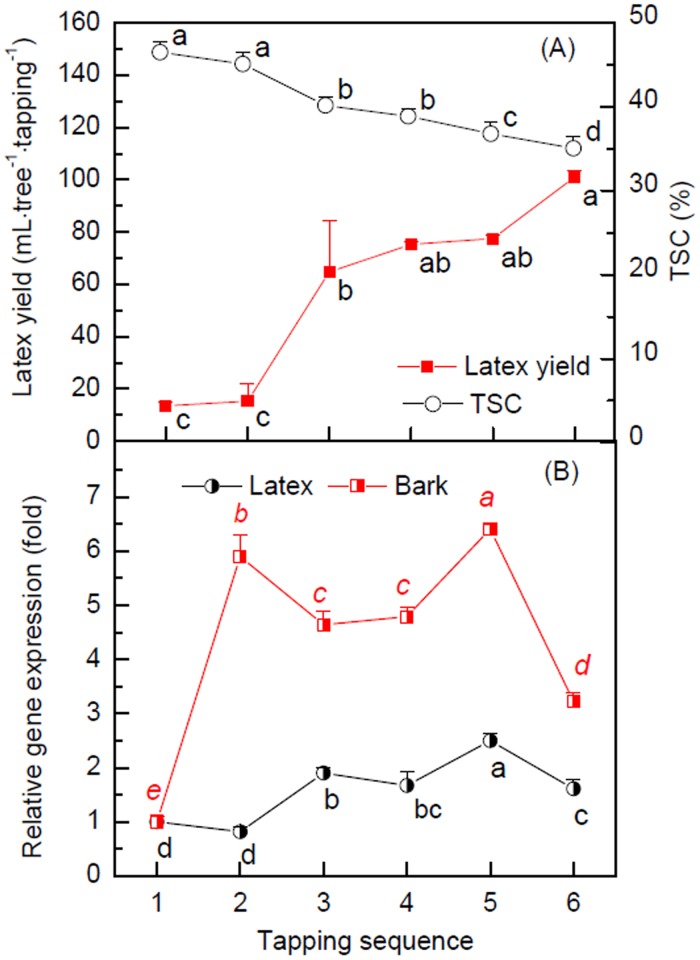
Effect of consecutive tappings on latex yield and *HbPIP2;3* expression. (A) Latex yield and total solid content (TSC), (B) *HbPIP2;3* transcript variation of virginal CATAS7-33-97 rubber trees subjected to six consecutive tappings. Data are means from three different trees and SD (n = 3). Different letters signify statistical differences between means at *P*<0.05.

The latex yield for a virginal rubber tree increases with successive tapping times before reaching its equilibrium at the sixth to tenth tapping [33, 54, 55]. This latex yield increase is normally accompanied by a corresponding decrease in TSC [33]. Fig 7A shows that the first and the second tappings of the mature virginal rubber trees CATAS7-33-97 produced only an average of 14.38 mL latex per tree per tapping. However, the stimulations from two tappings resulted in a significant surge in latex yield (64.75 mL/tree/tapping), an increase of 3-fold. This increase in latex yield continued until the sixth tapping. Coincidentally, the TSC started to decrease from the second tapping. There was a negative relation (y = 7.7563x+371.43, R^2^ = 0.9727) between the latex yield (y, mL/tree/tapping) and latex TSC (%). These results confirmed the concurrence of latex yield increase with TSC decrease in the wounding response. The further investigation of *HbPIP2;3* expression profile with tapping sequence (Fig 7B) showed that *HbPIP2;3* was significantly up-regulated 3.2 to 5.9 fold from the second tapping in bark and 1.6 to 2.4 fold from the third tapping in latex, which is consistent with the significant decrease in TSC and increase in latex yield from the third tapping. This result indicates that *HbPIP2;3* can be regulated by tapping and is involved in the tapping induced latex dilution and yield promotion during the first few tappings.

**Fig 8:**
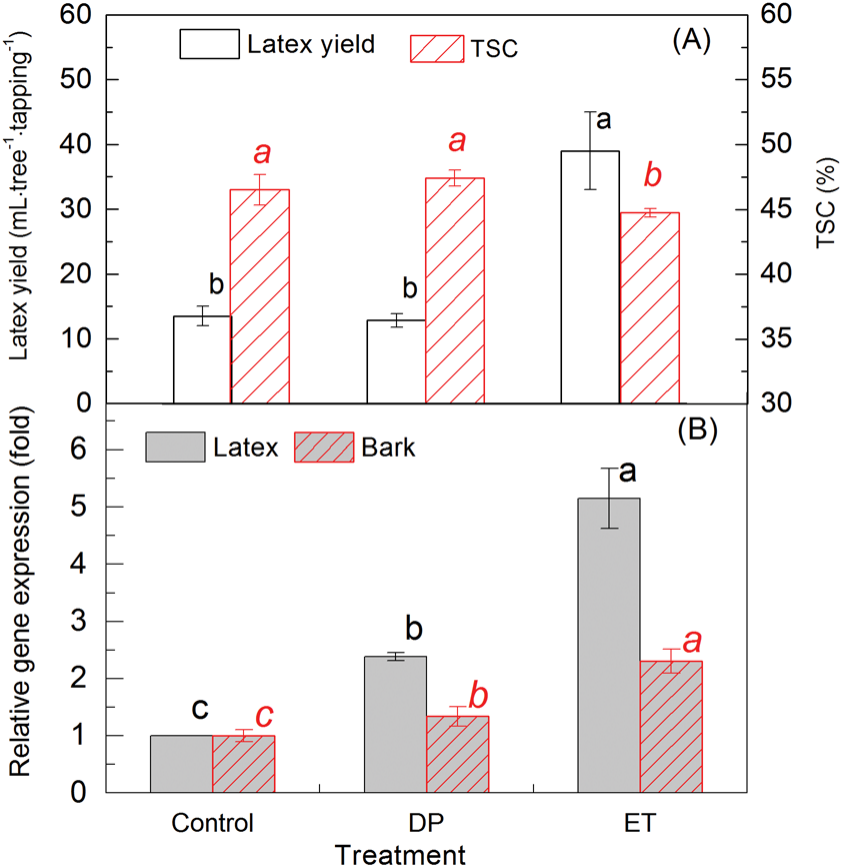
Comparison of wounding and Ethrel stimulation on latex yield and *HbPIP2;3* expression. (A) Change in latex yield and total solid content (TSC) and, (B) *HbPIP2;3* transcript expression in the virgin rubber trees (Control) and rubber trees subjected to drawing pin wounding (DP) and Ethrel (ET) treatments. The rubber clone was CATAS7-33-97. Data are given as means ± SD (n = 3) from three trees. Different letters indicate significant differences between means (*P*<0.05).

Tapping can cause both wounding induced sink effect and ethylene production [4, 37]. To eliminate the superposition effects of a tapping induced sink effect, wounding (without latex flow) and Ethrel treatment were applied to the CATAS7-33-97 virginal trees 24 h before the first tapping to better understand the impact of Ethrel on *HbPIP2;3* expression and latex yield. Although drawing pin wounding increased the expressions of *HbPIP2;3* in both the latex and the bark of virginal rubber trees by 2.3- and 1.3- fold (Fig 8B), the latex yield and TSC were not markedly changed (Fig 8A). However, the significant up-regulation of *HbPIP2;3* by 2.5% Ethrel stimulation in latex (5.2-fold) and bark (2.4-fold) resulted in a notable decrease of latex TSC and increase in latex yield. Therefore, although the drawing pin-induced wounding alone can up-regulate the expression of *HbPIP2;3*, it has marginal effects on *HbPIP2;3* expression and is inadequate to change latex TSC and latex yield. Tapping, wounding and Ethrel induced endogenous ethylene production is probably the underlying elicitor of *HbPIP2;3* up-regulation, and consequently the resultant latex dilution and yield promotion.

### Kinetic change of *HbPIP2;3* expression in response to Ethrel stimulation and its association with latex dilution and yield

**Fig 9:**
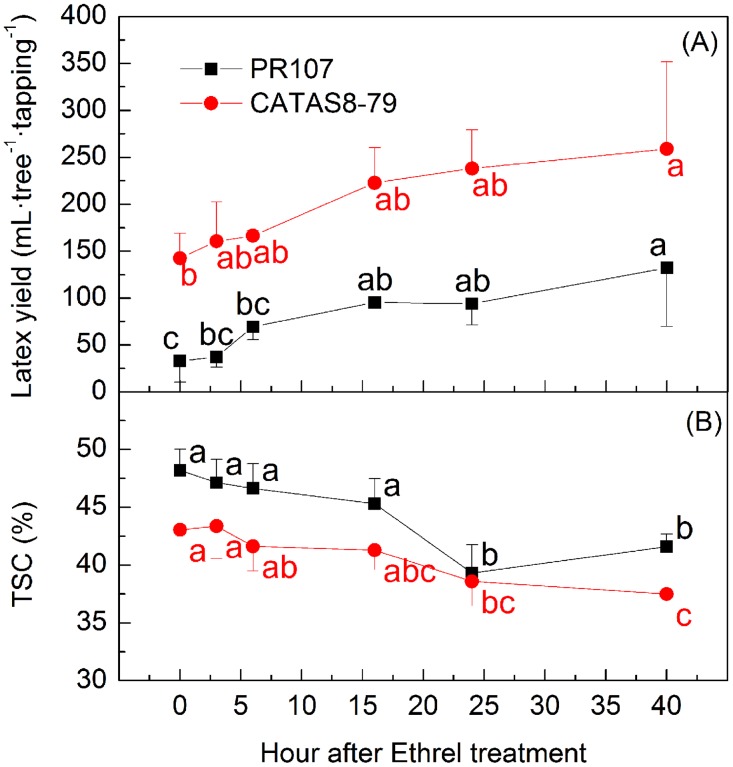
Kinetics of latex yield (A) and TSC (B) subjected to different duration of Ethrel treatment. The regularly tapped rubber clones with different responses to Ethrel stimulation were compared. The yield of PR107 responds well to Ethrel stimulation whereas CATAS8-79 rubber clone displays a relatively poor response to Ethrel stimulation. Data are means ± SD (n = 3) for each including three trees. Different letters represent latex yield or TSCs that are significantly different (*P*<0.05).

**Fig 10:**
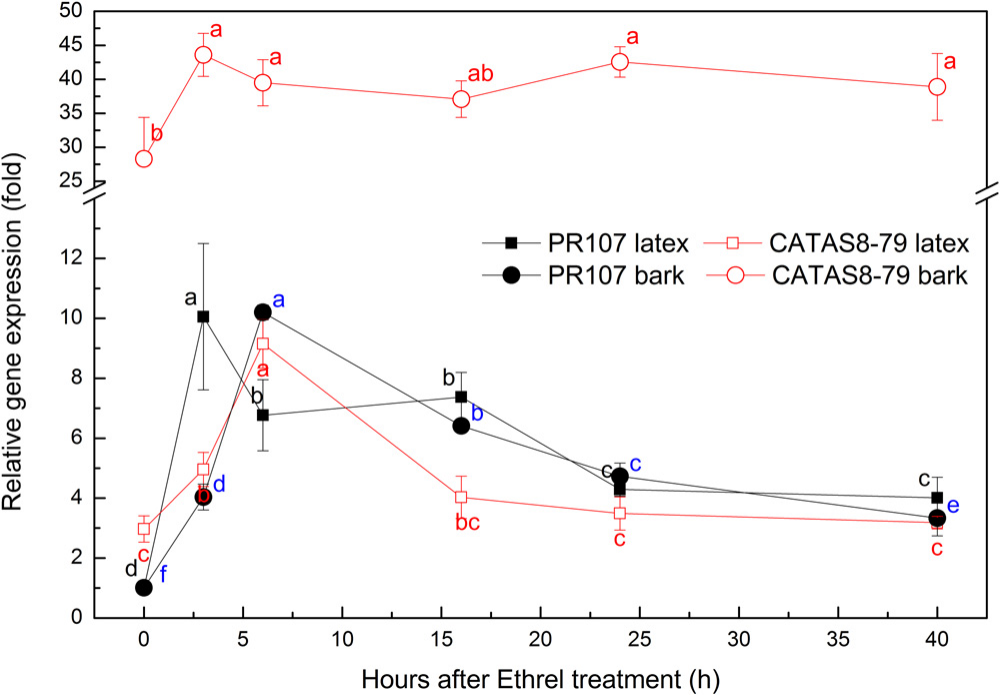
Variations of *HbPIP2;3* transcripts following Ethrel stimulation. The PR107 and CATAS8-79 rubber trees regularly tapped for three years were used as the plant materials. The reference gene was *HbYLS8* and the relative expressions of *HbPIP2;3* in the latex and bark samples of both PR107 and CATAS8-79 rubber clones were calibrated respectively by using the PR107 latex and bark samples without Ethrel stimulation (i.e. 0 hour after treatment) as the control sample. Data are means ± SD (n = 3). Different letters indicate that the transcript expression of *HbPIP2;3* was significantly different (*P*<0.05) among various treatment durations in the same clone.

Since Ethrel stimulation and tapping could both change the expression profile of *HbPIP2;3*, the kinetics of *HbPIP2;3* expression were investigated in regularly tapped rubber trees with different responses to Ethrel stimulation. As shown in Figs 9 and 10, Ethrel stimulation up-regulated *HbPIP2;3* expression, diluted latex and increased latex yield in both clones. For the Ethrel susceptible clone PR107, a significant increase in latex yield (up to 3- fold) and decrease in TSC (up to 9%) were observed from 24 h after the Ethrel stimulation (Fig 9). Correspondingly, the *HbPIP2;3* gene was up-regulated significantly as early as 3h and up to at least 40 h after treatment in both latex and bark. The up-regulation of *HbPIP2;3* by Ethrel treatment was 4- to 10-fold in bark and 3- to 10-fold in latex when compared to the control (0h, Fig 10). This up-regulation therefore facilitated the influx of water into laticifers and hence, dilutes latex and favours latex flow and latex yield.

However, for the clone CATAS8-79 which exhibits a low level response to Ethrel, the effects on latex yield increase and the latex TSC decrease were not as significant as PR107. The Ethrel stimulation up-regulated the gene expressions of *HbPIP2;3* by 1.5- and 3-fold at 3h and 6h after the treatment in the latex, and slightly up-regulated *HbPIP2;3* in the bark (Fig 10). Whereas, a relative low decrease in latex TSC and increase in latex yield can only be identified from 24h and 40h after Ethrel stimulation, respectively (Fig 9). This reduced response to Ethrel stimulation is probably because the expression of *HbPIP2;3* is initially higher in both the latex and bark, especially in the bark, of CATAS8-79 than in PR107 (Fig 10). The higher abundant of *HbPIP2;3* in CATAS8-79 than in PR107probably confers a lower latex TSC and higher latex yield in CATAS8-79. Therefore, *HbPIP2;3* is likely involved in rubber clone’s latex TSC, yield potential and Ethrel susceptibility.

## Discussion

### The involvement of *HbPIP2;3* in latex dilution and latex yield

Sufficient water supply to laticifer vessels is essential for both latex regeneration and latex flow [3, 4]. Within the rubber tree phloem, the concentrically arranged mature laticifers are devoid of functional plasmodesmatal connections [22], thus the exchange of water between laticifers and surrounding tissues relies largely on the PIPs, especially the PIP2 subgroup since the PIP1 subgroup generally exhibits no or poor water transport activity [9, 27]. Although two PIP2s have been previously cloned from rubber trees, it is likely that more PIP2 genes remain to be cloned. Other species have been found to have more than twice that found for rubber tree so far, for example, *Arabidopsis thaliana* and rice both have 8 PIP2 aquaporins [9]. In this study, the highly expressed and Ethrel-regulated aquaporin *HbPIP2;3* identified from the latex transcriptome has been cloned and functionally characterized. *HbPIP2;3* shares a high similarity with the homologs from other plants and the previously reported *HbPIP2;1* [23]. Similar to the previously identified *HbPIP2;1*, *HbPIP2;3* encodes an efficient water transporter that has been validated using *X*. *laevis* oocytes. However, unlike *HbPIP2;1*, the water transport activity of *HbPIP2;3* can be effectively inhibited by HgCl_2_ as observed for many other plant aquaporins [50–53]. The Hg-sensitivity is potentially conferred by the Cys-95 residue that is present adjacent to the first NPA motif [56] or the Cys-131 harboured in the third transmembrane helix [57] of its deduced amino acid sequence (Fig 2).

In the dominant PIPs identified from the rubber tree latex transcriptome, *HbPIP2;3* is the most highly expressed functional aquaporin that is up-regulated by Ethrel (Figs 1 and 5), suggesting its crucial role in rubber tree phloem water relationships and Ethrel induced latex dilution. Its greater expression in latex compared with that in any other tissues implies a prominent role in water influx into laticifers. The concomitance of rubber tree latex yield increase with latex TSC decrease and with *HbPIP2;3* up-regulation (Figs 7–9) has confirmed the importance of *HbPIP2;3* in rubber tree latex dilution and yield.

Nevertheless, the role of the most abundant but water channel inactive aquaporin, HbPIP2;7 [49], in laticifer water balance and latex production cannot be ruled out. Although all plant PIP2 proteins reported, with an exception of PttPIP2.1 and PttPIP2.2 [58], have been found to have high water channel activity when expressed in *Xenopus* oocytes, the heterogonous expression of aquaporins cannot always be successfully inserted into oocyte plasma membranes [5]. The localization of this gene with GFP (Green-fluorescent-protein) fusion proteins or immunodetection is required to confirm the negative result. Meanwhile, while swelling assay of aquaporin function in *Xenopus* oocytes is commonly employed in plant research, the swelling behaviour of *Xenopus* oocytes does not necessarily reflect the transport property of aquaporins in plant [58]. In addition, besides water, emerging evidence showed that aquaporins could also facilitate the permeability of some neutral molecules (for example, glycerol, and urea), gases (such as carbon dioxide), ammonia, hydrogen peroxide, metalloids, silicon, boron, arsenite and antimony [9, 59]. There are also synergistic activation and interactions among aquaporins [11]. If HbPIP2;7 is indeed not functional for water transport, it might be involved in other processes.

### The regulation of *HbPIP2;3* by tapping, wounding and Ethrel stimulation and its relationship to latex yield

The increase in latex yield and water content following the first few successive tappings of a virginal rubber tree has been well documented [33, 54, 55] and has been referred to as a wounding response phenomenon [60]. However, the reason for the concomitant decrease of latex TSC with latex yield increase has not been studied. In our study we have confirmed the direct association of latex yield increase with TSC decrease on the mature virginal CATAS7-33-97 rubber tree clones. We further found that tapping can up-regulate the expression of *HbPIP2;3* which contributes to latex dilution and yield promotion in the wounding response. Tungngoen et al. [23] has indicated that the induced-expression of the *HbPIP2;1* aquaporin gene results in latex dilution in virginal PB217 rubber trees because it facilitates laticifer water exchange. We propose that *HbPIP2;3* plays a similar role in rubber tree clone CATAS7-33-97 in regulating the water content of latex, which is a determinant of latex fluidity. It is hence very important to identify the aquaporins that contribute to latex dilution and consequently, the means by which they are up-regulated.

Successive tappings up-regulates the expression of *HbPIP2;3* aquaporins. However, a tapping is actually a form of controlled wounding that included the removal of a thin shaving of bark and phloem from the surface of the previous tapping cut. Tapping results in two interacting effects near to the tapping cut: the sink effect resulting from the exudation and regeneration of latex, and the wounding effect which may induce ‘wound ethylene’ release and endogenous ethylene accumulation [4, 37, 61]. By comparing wounding alone (without latex exudation) with Ethrel stimulation alone on the virginal rubber trees where there was no tapping induced sink effect superposition, we found that *HbPIP2;3* was up-regulated by both wounding and Ethrel stimulation alone. The up-regulation of *HbPIP2;3* can thus be ascribed to the endogenous ethylene induced by both wounding and exogenous Ethrel application. This result is consistent with the up-regulation of *Vitis vinifera* aquaporins [62] and *HbPIP2;1* [23] by ethylene and the up-regulation of *PatPIP1* by wounding in *Populus alba × P*. *tremul*a var. *glandulosa* [63]. By screening the 5'-flanking region of *HbPIP2;3* from the rubber tree genome [39], an AATTCAAA box, which was previously shown to be an ethylene responsive element in the promoter region of many ethylene-induced genes [64–66], was identified at the site of -1627(unpublished data). Therefore, it is not surprising that *HbPIP2;3* can be regulated by ethylene and contributes to latex dilution and yield promotion in *H*. *brasiliensis*.

Our results have shown an obvious link between an increase in latex yield and water content following Ethrel application. The kinetic study of *HbPIP2;3* expression with Ethrel stimulation on regularly tapped rubber trees showed that *HbPIP2;3* was markedly up-regulated as early as 3 h after Ethrel application and high up regulation continued for more than 40h. This up-regulation occurred prior to the significant decrease of latex TSC and increase of latex yield, and fits with the molecular events that normally precede physiological modification [23, 67]. The expression pattern of *HbPIP2;3* in the PR107 and CATAS7-33-97 clones that were regularly tapped is consistent with the expression profile of *HbPIP2;1* in the PB217 virginal tree [23]. Also, it is supported by the research of Tupy [68] that showed that tritium water intake into laticifers was markedly accelerated within 4 h after Ethrel stimulation. By considering all these effects, it can be concluded that tapping and Ethrel-induced endogenous ethylene can quickly up-regulate the expression of aquaporins and thus favour latex dilution and production of a rubber tree. Even though tapping alone could induce an up-regulation of *HbPIP2;3* in the first few tappings, its expression profile following Ethrel stimulation in the regularly tapped tree exhibited similar kinetics to *HbPIP2;1* in virginal rubber trees [23]. The combination of these results that show up-regulation of aquaporins with those that showed up-regulation of sucrose transporters [37, 69, 70] and a quebrachitol transporter [71] upon Ethrel stimulation partially explains the mechanism of tapping- and Ethrel stimulation-induced latex dilution and yield promotion.

### The relationship of aquaporin expression with rubber tree clone susceptibility to Ethrel-induced yield promotion

Various rubber tree clones respond to Ethrel stimulation differently [4, 33]. Rubber tree clones with low TSC normally exhibit high rubber yield potential but limited response to Ethrel [2, 29, 32, 33]. However, the relationship between rubber tree latex TSC and the Ethrel stimulation response and aquaporin expression has not been reported. In this study, the latex yield, TSC and *HbPIP2;3* expression profiles were compared in the rubber clones that had different responses to Ethrel stimulation. The latex of metabolically active clone CATAS8-79 has a lower TSC and a higher yield than the low metabolic clone PR107 since the initial expression of *HbPIP2;3* in CATAS8-79, especially in the bark, is correspondingly higher than that in PR107. In addition, due to the higher basal expression of *HbPIP2;3* in the CATAS8-79 clone, Ethrel stimulation had a limited effect on *HbPIP2;3* expression compared with that of clone PR107. Therefore, in response to Ethrel stimulation, the decrease in latex TSC and increase in latex yield by the CATAS8-79 rubber clone is limited when compared to the PR107 rubber clone. It has been suggested that the regeneration of rubber particles will be accelerated at a low latex concentration [3]. The results of our study suggest that PIP aquaporin expressions appears to be linked to the clonal characteristics of rubber tree latex TSC, yield potential and Ethrel stimulation response. However, as the comparisons were made only on the regularly tapped trees of clones PR107 and CATAS8-79, further investigations are required to fully understand this relationship. Meanwhile, it is suggested that aquaporins are involved in phloem turgor pressure regulation [72] and translocation [73]. The mechanism of how aquaporin expression is associated with rubber tree phloem turgor pressure and sucrose translocation and ultimately latex yield warrants further study.

## Conclusions

A novel gene *HbPIP2;3* encoding an efficient water transporter was identified from the rubber tree. The gene is highly expressed in laticifers and its transcripts can be up-regulated by wounding, tapping and Ethrel stimulation. Furthermore, the transcriptional level of *HbPIP2;3* is closely related to latex TSC and latex yield, indicating its involvement in latex dilution and latex yield promotion. The expression kinetics of *HbPIP2;3* in the regularly tapped rubber tree had similar kinetics to *HbPIP2;1* in the virginal rubber trees. It is now apparent that PIP aquaporin expression is linked to the clonal characteristics of rubber tree latex TSC, yield potential and Ethrel stimulation response.

## Supporting Information

S1 File The PIP aquaporins identified from rubber tree latex transcriptome. **File A**, The nucleotide sequences of ten transcripts identified in the rubber tree latex transcriptome. **File B**, Phylogenetic analysis of identified HbPIPs with the complete set of *Arabidopsis* PIPs encoded in the genome.
(PDF)

S2 File The nucleotide sequence and gene structure of *HbPIP2;3*. **File A**, The transcriptional region of *HbPIP2;3* gene and its deduced coding protein. **File B**, The schematic gene structure of *HbPIP2;3*.
(PDF)

S1 Table Gene specific primers used for quantitative RT-PCR assay of aquaporin expressions in rubber tree latex. (DOC)
