# Supplementary material for: Regulation of HbPIP2;3, a Latex-Abundant Water Transporter, Is Associated with Latex Dilution and Yield in the Rubber Tree (Hevea brasiliensis Muell. Arg.)
Source: PLoS One. 2015 Apr 30;10(4):e0125595. doi: 10.1371/journal.pone.0125595 (PMC4416032; doi:10.1371/journal.pone.0125595)

**S1 File. The PIP aquaporins identified from the rubber tree latex transcriptome**

**File A.** The nucleotide sequences of ten transcripts identified in the rubber tree latex transcriptome. The start and stop codons in each transcript are blacked.

>HbPIP1;1 Unigene5144

CACTTATCAAAATCAAACGGCCATTAACAAGCGTCTGATCATCAATTTCAGTGTATTGGCTTTGGTCTT
TTTGGAGCTAAACAGCT**ATG**GAGGGCAAGGAAGAAGATGTTAGATTGGGAGCTAACAAATATAGG
GAGACGCAGCCCATTGGTACGTCAGCTCAGAGCCAAGATGACAAGGACTACACTGAACCACCAGCA
GCGCCGCTGTTCGAGCCAACGGAGCTCACTTCTTGGTCCTTTTACAGGGCTGGTATTGCAGAGTTCAT
AGCCACTTTCTTGTTCTTATACATCTCTGTTTTGACTGTCATGGGTGTAGTTAAGGCACCCACCAAGTG
TTCGACTGTTGGGATTCAGGGGATAGCTTGGTCCTTTGGTGGCATGATCTTCGCTCTTGTCTATTGTA
CTGCTGGCATTTCAGGAGGTCACATAAACCCGGCGGTGACGTTTGGGCTGTTTTTGGCAAGGAAACT
GTCCTTGACAAGGGCTTTGTACTACATGGTGATGCAGTGCCTTGGAGCCATATGTGGTGCTGGTGTA
GTGAAAGGATTTGAAGGGCGTCACCAGTATACTTTGTTGGGTGGTGGTGCCAATAGTGTGAACCCA
GGTTACACCAAAGGTGATGGGCTTGGTGCTGAAATTGTTGGCACCTTTGTTCTTGTCTACACTGTCTT
CTCTGCTACTGATGCCAAACGTAATGCCAGAGACTCCCATGTTCCTATTTTGGCACCTTTGCCAATTG
GGTTCGCTGTGTTCTTGGTGCACTTGGCTACCATCCCAATTACAGGAACTGGTATCAACCCAGCTCGT
AGTCTTGGTGCAGCAATCATCTTCAACAAGGACAAGGGCTGGGATGATCACTGGATTTTCTGGGTGG
GTCCATTCATTGGAGCAGCACTTGCAGCTCTATACCACCAAGTTGTGATCAGAGCCATCCCTTTCAAG
AAG**TGA**TCATGATGCAAATGTTTATGGCCATTTCTTGTTTTCAAAATCAAGACCAAGATCGAGCTCAG
TTTGTTAGCTTGTTTATCTGTTTCATTACTGTGTACTTTTAACCATATGAGCACCCTGTAATAATAGCAC
TATGTTTTCCTTGTCAAAGAGCCTTCTTCTACCTTCCTTTTTATCTGTGGAAGGGAAGGAAAGTGGGC
CATGTCTGATGAGCCCATCTTAAGTTAGGTGGGCTGTATGTATTCCTATGATGGTATTTATGTGAATG
GTTGTGAATTTCTGGAATGTCATTTTCTTTGGCTCCGACCAAAAACCTACTTCTTAGCTCATGCTCTAA
AAGAGTACAATAATTGATAGTGCCAAGTTGTCCTTTTTCCCCGTTTATCTTGTAATTATTAATGTTGTG
TGAGGTGGGATAAGGCAGCTTTTGATGATTTGGCTTTGCTTCCTTCCATTTTTTTCCCTTCCCCTCTCTA
GAATATTGAATTTCTTTAGTTTCTTACTCTAGCTTGGAGATAAGCTTTATTATTGCGACTAATCTACGA
GATAATATACAAAAATTCCT

>HbPIP1;2 Unigene48226

ACTGATAAAAAATGAACGGTCACTAACAAGCCTTTGATCAAGTTTCAGTATCTTGGTGTTTCTTGAGC
TTAAACAGCT**ATG**GAGGGCAAGGAAGAAGATGTTAGATTGGGAGCTAACAAATATAGGGAGACGC
AGCCCATTGGTACGGCGGCTCAGAGCCTAGATGACAAGGATTATACTGAGCCGCCACCGGCGCCGC
TATTTGAGCCGACTGAGCTCACTTCTTGGTCCTTTTACAGGGCTGGTATTGCAGAGTTCATAGCCACT
TTCTTGTTCTTGTACATCTCTATTTTAACTGTGATGGGTGTGGTTAAGGCACCCACGAAGTGTTCAACT
GTTGGGATTCAAGGGATCGCTTGGGCCTTTGGTGGCATGATCTTCGCTCTTGTATACTGTACTGCTGG
CATTTCAGGAGGTCACATAAACCCGGCGGTGACGTTTGGGCTGTTTTTGGCAAGGAAACTGTCCTTG
ACGAGGGCTTTGTACTACATGGTGATGCAGTGCCTTGGAGCCATATGTGGTGCTGGTGTGGTGAAA
GGATTTGAAGGACGTCACCAGTATACTTTGTTGGGTGGTGGTGCCAATAGTGTGAACCCAGGTTACA
CCAAAGGTGATGGGCTTGGTGCTGAAATTGTTGGCACCTTTGTTCTTGTCTACACTGTCTTCTCCGCC
ACTGATGCCAAACGTAATGCCAGAGACTCCCATGTTCCTATTTTGGCACCTTTGCCAATTGGGTTCGC
TGTGTTCTTGGTGCACTTGGCTACTATCCCAATTACAGGAACTGGTATCAACCCAGCTCGCAGTCTTG
GCGCAGCAATCATCTTCAACAAGGACAAGGGCTGGGATGATCATTGGATTTTCTGGGTGGGTCCATT
CATTGGAGCAGCACTTGCAGCTCTGTACCACCAAGTTGTGATCAGAGCCATTCCTTTCAAGAAG**TGA**
TCATATGATGCATATATGTGGCCGTTTGATTATGTTTTCCCGATCAAGATCAAGATCAAGATGAAGCT
CAGTTTGTTTGCATTTTCTATGTTATATTACTTTGTATTTGTACCTATATGAGCACATATAATAATAGTA
TTATGTTTCCTACCAAGGGCCTTCTTCTACCTTCTTCCTTTTTTTTTATTTGTGGAAGGGAAGAGGTGG
GTGACGAGTGCTGGGCCCATGTTAAGTAGGTGGGCTGTATGTATTCATATGATAATGTTTATGTGAA
TAGTTCTGAATTGTGAAATGTCATTTTCTTGTCAGCCGACCGAAAACCTACTCTCTTAC

>HbPIP1;3 Unigene13477

ATTGGGTTAAGGCTCAACAATCAATGACGCTTTGCTATCTACAAAATGCCCTTATCTTCTACGCCCTCG
CAGGCTCTGAAGCTTTTACTTGCCTTTTCAGTGTGCTTTAACTCTACTTAGAGAGGGTCAAGAATCCA
TCTTTTTTCTGTGGGAGAGAGAGACGGATAGAGCA**ATG**GAGGGCAAGGAAGAGGATGTTAGACTA
GGAGCAAACAAATTCACAGAGAGGCAGCCCATAGGGACATCAGCCCAGACTGATAAGGACTACAAG
GAGCCACCACCAGCTCCCCTGTTTGAGCCAGGTGAGCTATGCTCATGGTCCTTCTACAGGGCTGGGA
TTGCCGAGTTCATCGCTACTTTCTTGTTCCTCTACATCACCGTATTGACTGTCATGGGTTTCTCTAAGTC
CACCAACAAGTGTGCCACTGTAGGTACCCAGGGTATTGCTTGGGCCTTTGGTGGTATGATCTTTGCCC
TTGTCTACTGCACTGCTGGTATCTCAGGTGGACACATCAACCCAGCAGTGACCTTTGGTCTGTTTCTG
GCAAGAAAGCTCTCCTTGACAAGGGCTCTGTTCTACATCATCATGCAATGCCTTGGTGCCATCTGTGG
CGCTGGGGTGGTGAAAGGTTTTGAGGGAGATCGTGTATATGAGACTTTGGGTGGTGGAGCCAACGT
TGTGGCTCATGGCTATACTAAGGGTGATGGTCTTGGTGCTGAGATTGTCGGCACCTTTGTTCTTGTCT
ACACTGTCTTCTCTGCCACCGATGCAAAGAGGAACGCCAGAGATTCTCATGTCCCTATTTTGGCTCCT
CTTCCCATTGGGTTTGCTGTGTTCTTGGTTCATTTGGCCACCATCCCCATAACTGGAACTGGCATTAAC
CCAGCCAGGAGCCTTGGAGCTGCTATCATCTTCAACAAAGACCACGCATGGGATGACCATTGGGTTT
TCTGGGTTGGGCCCTTCATTGGAGCTGCCCTTGCTGCCGTGTACCACCAGATAGTCATCAGAGCTATC
CCTTTCAAGGCCAGAGCT**TAA**TGTTTTTACCATCTTAGTCATCAGAGGCCGCCTTTGAGTCTTATCAAA
CACTTCTTCCTTGGTGTTCTCCTCTTCATTTCTTTTGTTGTGTATTTTTGTTTCTATCCCTCCATGTGAAT
CTGGTATTTGGAGTGTTATCACGAGTGTAAATTATCTAGTGTTGCTGTATTATTGTGATACCCACTGA
ATGCAGAAAGCCCTTTTATCTTTAATCTTTGCTTTTTCCTAAGCAAATCTTGTC

>HbPIP1;4 Unigene646

GAGAGGATCAAAAACCTTTTCTTTTCTTGTGTGAGGTGAGGAGAGAGAGAGAGAGATAGAGAGAG
AGTGAGAGAGAGCA**ATG**GAGGGCAAGGAAGAGGATGTTAGACTAGGAGCAAACAAATTCACAGA
GAGACAACCCATAGGGACATCAGCCCAGACTGATAAGGACTACAAGGAGCCACCACCTGCTCCCTTG
TTTGAGCCAGGTGAGCTTAGCTCATGGTCCTTCTACAGGGCTGGGATTGCCGAGTTCATCGCTACTTT
CTTGTTCCTCTACATCACTGTCTTGACTGTCATGGGTTTCTCCAAGCCAAATAACAAGTGCACCACAGT
GGGTACCCAAGGTATTGCTTGGGCCTTTGGTGGTATGATCTTTGCCCTTGTCTACTGCACTGCTGGTA
TCTCAGGTGGACATATCAACCCAGCAGTGACCTTTGGTCTGTTTCTGGCAAGGAAGCTCTCCTTGACA
AGGGCTTTGTTCTACATGATCATGCAATGCCTTGGTGCCATCTGTGGTGCTGGGGTGGTAAAAGGTT
TTGAGGGAAACCGTGCATATGAGACTTTGGGTGGTGGAGCCAACGTTGTGGCCCATGGCTACACTA
AGGGTGATGGTCTTGGTGCTGAGATTGTTGGCACCTTTGTTCTTGTCTACACTGTCTTCTCTGCCACT
GATGCAAAGAGGAACGCCAGAGACTCTCATGTCCCTATTTTGGCTCCTCTTCCTATTGGGTTCGCAGT
GTTCTTGGTTCATTTGGCAACCATCCCCATCACTGGAACTGGCATTAACCCAGCCAGGAGTCTTGGGG
CTGCTATCATCTTCAACAAAGACCACGCATGGGATGACCATTGGATTTTCTGGGTTGGACCCTTCATT
GGAGCTGCCCTTGCTGCTGTGTACCACCAGATAGTCATTAGAGCTATCCCTTTCAAGGCCAGGGCC**T**
**AA**TGTTTTAGCATCACTTTGCTCCCGGTGTTCTCCTCATCATTTCTTTTTATTATGTTACTTTGGTTTTTG
TCCCTCCATGTGAATCTGGGTATTGGAGTGTTAATTATGTGTGTAAATTATCTAGTGGTGCTGTATTA
AGCATTGTGATGCCTATTAAGCATTGTGATGCCTATTGAATGCAGAGATTCTTTTTTACTTATAACCTT
GCTCTCTCTCACTCTCTCTCTCTCT

>HbPIP1;5 Unigene33127

GATGATGTTGAGAACATATTATACCAGGGCAGCCCATAGAAAAGAGGGAACTGTTGCGTTGTGGGT
GCCCACTTTGCGTGATACACATTAGCCATGTGTTGGGATCCACGGTACTGCTCGAAGCTTACTCATGC
AACGCATTACTCATGGGCGGCTAATCCAGCCAGCAGACCCAAGTCACCTTCTTCCGCCGAAACCACAT
ACGCAGCACCATATCCTGCTGAAAGCAAATTGAAGCGAGAAAAAATGGAGGGGAGAAAAGGATGT
GAGGTTAGGAGCAATCAAGTTCCAAGAG**ATG**CAACCCCTAGGTACCTCAGCCCAGACAGACAAGGA
CTACAGGGATCCACTACCAGCACCTCTCTTCGAGCCTGTTGAGCTCAAGTCCTGGTCTTTCTGGAGAG
CTGGAATTGCTGAGTTTTTCGCCACCTTTTTTTTCCTCTATGTCACTGTATTGACCGTCATGTGCTTCAG
CAGGTCACCTAATAAGTGTGCCTCTGTGGGTGTCCAGGGCATAGCTTGGGCCTTTGGGGGCATGATA
TTTGTTCTTGTTTATTGCACTGCTGGTATCTCAGGTGGGCACATAAACCCTGCAGTAACTTTTGGTCTT
ACCTTGGCAAGGAAGGTGTCCCTCACCAGAGCCATATTCTACATGGTGATGCAGTGCCTTGGAGCGA
TCTTCGGAGCAGGGATTGTGAAGGGGTTTCAGCCAACACCGTTTGAAACGCTGGGTGGTGGAGCTA
ACGTGGTAAACCCAGGGTATTCCAAGGGCGACGGCCTTGGTGCTGAGATTGTTGAAACCTTTGTGCT
TGTCTACACTGTTCTCTCTGCCACTGATGCCAAGAGAAGCGCTAGAGACTCCCATGTCCCTATACTGG
CATCACTCCCTATTGGGTTCGCAGTGTTTTTGGTTCACTTGGCCACCATACCCATTACAGGAACTGGA
ATCAACCCTGCTCGCAGTCTTGGAGCAGCAATCGTATACAACGAGGACTGTGCATGGTATGATCATT
GGATCTTCTGGGTACGACCTTTCATTGGCGCTGCGCTTGCTGCACTATCCAACCAGATAGTCATCAGA
GCCATCCCATTTATGTCCAAAGCC**TGA**GAACCAACTTTCACAATCTTTTTATGTTCTGTTTATTGTATA
GTTATGTTTTATGTCTTCTTCCTCCCTCTATTTTCAAGTTAACATCTTTAAGAGTTTAAATGTTAATTAT
GTGTATGAGCAACTGTTCTAAATGCATGTTCGAAACGAATCCAGTACCATTAATTAAAAC

>HbPIP2;1 Unigene30012

GGAAGTGGTTGGGGGAATCAACGGTGGTGTTATAGTTGGACTTGAGTTGAATGGACGGTTATTGGT
GGCCATATGAATGGTGATATTGTTTATGGACGGTTTTGTCGCCATTACCCACCACGTCATTCCCTCAC
ATTAGTCAATAAAATGGTGGGTCCACCCGCATACCATCTTGGAGATACGTGCTATCTCATAAGCTACT
ATACTTGTTCCGCCCAGAGTAAAACAACGTCGTATTAATACCAGCCCATGGCACTTCATTTCTTGGAA
AATTGCATGTGTAAGTTGGATTATGCAACCATTCATGCGGAATGTTAGGTTAAAAGTTCTCCAATGTT
CGCAAGAAAATGTGGCCATAATTTCTTGGCGATTGGAGAATGCAAGTAAAACCAATCACATGGAAA
GGCCACAGCAGTTGCCAGAATTGAGAGACTATTAGAACTTACCCGAACAATCTCTACTCTTTAGCCGA
ATCAGAAGAGGCCAAAGAAGCAATCACATGAAGTGTGAGGGTGGGGCCACAGAAAGCGCAAGAAA
CCATTTGTTAAGTTAGATGGGGCGGCAAAGCTAAGTTAAATTAACACTCACTCCTCTCAAAACTCTTC
AATACTCAATACTCACATATCCCATAAATACCCCCCTCCACTTCCACACTTCCCACTACAATAATCAAC
ATCTCTTAGCTCAGTCGTCTCTTTCCTTTCTCTTGCCAAATTCTGTCACGGTACCT**ATG**GCCAAGGACG
TTGAAGTTGGAGGACAAGGCGGTGAGTTCCAAGCCAAGGACTACAATGACCCGCCACCTGCACCAC
TGATCGATGCCGAGGAGTTTACTCAGTGGTCGTTTTATAGGGCTATCATTGCTGAGTTTATAGCCACG
CTTTTGTTTTTGTACATCACTGTTTTGACTGTGATTGGTTACAAGAGCCAAACCGACCCTGCCAAGAA
CGCTGATCCATGTGGTGGTGTTGGGATTCTTGGTATCGCTTGGGCCTTCGGTGGAATGATCTTTATTC
TTGTTTACTGCACTGCTGGTATCTCAGGTGGGCATATAAACCCTGCTGTGACTTTGGGTCTGTTTTTG
GCGAGGAAAGTGTCTCTGGTGCGAGCTATATTGTACATGGCAGCTCAGTGCTTGGGAGCCATATGT
GGATGTGGGCTCGTGAAAGCATTCCAAAAGGCTTATTACAATAGGTATGGTGGTGGAGCCAATGAG
CTTGCTGATGGGTACAGCAAGGGCACTGGATTGGGTGCTGAGATCATTGGTACCTTCGTTCTTGTCT
ACACTGTCTTTTCTGCTACTGATCCCAAAAGGAATGCTAGAGACTCCCATGTTCCTGTCTTGGCTCCAC
TTCCAATTGGATTTGCTGTGTTCATGGTTCATCTGGCCACAATTCCAGTCACTGGCACTGGTATTAACC
CAGCTAGGAGCTTTGGAGCTGCTGTGATCTACAACCAGGACAAGGCATGGGATGACCAATGGATTT
TCTGGGTTGGACCTTTCATTGGTGCCGCAATTGCTGCATTCTACCACCAATATATCTTGAGAGCAGCT
GCTGTTAAAGCTTTAGGATCTTTCAGGAGCACGTCCAACATA**TAA**TTGAGAAAAGAAATTCCTTTATG
CTACTAAATTTGAGAGTTTCATCTGGTCCTTGAGGCTTTGGGACCTTGGAATATGTACATTAATCATG
TATCTTTGTGTGCTTGGGTTCATGGTTTTATTTGTGCCCAAACTGTTGCTTCCACTCTATTTTCTTTTTCC
CCTTTGTGGCCTTGCACTCATGATGATGTAGACAAATTTATGAGCATTGTGTCAATTATATGATTTCAA
TTGGAAAATAAAATTCTTATATATTAATATATGCCATTGATGAAGAGATGTTGATCATATTCAATAGA
TTGAGAG

>HbPIP2;3 Unigene687

AAAAATTGTTTCTCAATATTAAATGGAAAAATGACTAAATAAGTACTTTATGAAATAAAAAATAAAAT
CCTTGGAAACAAAGAAAATGAAAAAAAAAGAAAAAGAAAAATGAAAAGAAAACAGCCTTTACTTTA
CCCGCTCTCCTTCACCATTTATACACTCGCTCAGCTCAGCTTCTCTGTATCCTAGGTCTCAAAACCAAA
ACGCCAACCCCCTCTCTCTCCCTCTCTATCCCCTTATATACAAAATCTTGCTGTTTCTCTTTGCTGAGGG
TCGACC**ATG**GTGAAGGACGTGACAGAACAAGGATCATTCCCAGCGAAGGACTACCATGACCCACCA
CCAGCACCATTGATTGATGCGGTGGAGCTAACCAAGTGGTCATTTTACAGGGCCTTGATTGCTGAAT
TTATAGCAACTTTGCTCTTTCTTTACATCACTGTTTTGACTGTGATTGGATACAAAAGCCAGACTGATC
CTGCAAAGAATGCTGACTCTTGTGGTGGTGTTGGAATTCTTGGCATCGCTTGGGCCTTTGGTGGCAT
GATCTTTATTCTTGTTTACTGCACTGCTGGTATTTCAGGAGGGCACATTAACCCAGCAGTGACATTCG
GGTTGTTCCTGGCCAGAAAGGTCTCACTGGTACGGGCCGTCATGTACATGGTGGCCCAGTGCTTGGG
AGCCATAGCCGGTGTCGGATTGGTGAAGGCCTTCCAGAGTTCCTTCTATAAGAGGTATGGTGGTGG
GGCCAACAGTCTGGCTGCTGGGTACAGCAAAGGCGTTGGATTGGGTGCCGAGATCATCGGGACTTT
TGTTTTGGTCTACACAGTGTTTTCCGCCACAGATCCGAAGAGGAATGCCAGAGACTCCCATGTGCCG
GTTTTGGCTCCACTCCCAATTGGATTTGCTGTATTCATGGTTCACTTGGCCACCATTCCAATCACTGGA
ACCGGCATCAACCCAGCCAGGAGTCTAGGAGCTGCTGTTATCTACAATCAGGACAAGCCCTGGGATG
ATCATTGGATCTTTTGGGTTGGTCCCTTCATTGGTGCAGCCATTGCAGCCTTCTATCACCAATTCATCT
TGAGGGCAGGAGCTGTGAAGGCTCTTGGGTCATTCAGGAGCAACCCAACTGTT**TAA**GGGAAGAAAT
AATTTTTCTAAACTAATAATGAAGGAAAAGCATGTGCTGGTTGGTTTCATTATTAGCCCTTCTGGTGT
GTTCTTTGTTGTTGGGGGGGTTGAAAATGTGAGAAGAGAGGTTTGAAAGAATTATGGAATTTGTAG
ATATAAAAGCCTCTTTGAAAATGGGAGGCTTTTGGTTATCCCTTTTTTATTTGTTTTGTTTGAGTTCAC
TTAGCAATGTTATGATGGTTTTCTTGGTTGGTTCATCATCATCATCTATTGTCTATTTTTTCTTCTTTGC
ACTTATTATGCTTGTAAGAAAAGTTAGGCTCCTTTGCTTTTATTATAAGTGTAGGCTATGCTTGTTTTA
TTATGAGCTTTAAGTTTCATTTTCACAAAAAAAAA

>HbPIP2;4 Unigene15267

ATAAATACCCCTCTGTGCTCGCCTCTCCAACCGCACATTCTCCACTCTCTCTCTCTCTCTCTCTCTCTC

TCTCTCTGAGCTCACTAGCTACCAGAGAAGCAGCAAATCAAGAAAAGCTGTAGAGCAATAATTGTCT

C**ATG**GCTAAGGACGTGGAAGTTGCAGAGAATCCTGGAGAGTTCTCAGCCAAGGACTACCATGACCC

TCCACCTGCACCTTTGATCGATGTTGAGGAACTTGGAAAATGGTCCCTCTACAGAGCTCTTATTGCAG

AGTTCATAGCCACCCTTCTCTTTCTTTATATCACTGTTTTGACAGTTATTGGATACAAGAGCCAAACTG

ATCCTCTCAAGAATGCTGATGCTTGTGGTGGTGTTGGTATTCTTGGTATTGCTTGGGCCTTTGGTGGC

ATGATCTTTATTCTCGTTTACTGCACTGCTGGTATCTCTGGAGGACACATTAACCCAGCTGTGACCTTT

GGGCTTTTCTTGGGACGCAAGGTGTCACTAATCAGGGCCCTTTTATACATGGTAGCACAGTGCTTGG

GTGCAATCTGTGGGTGTGGTTTAGTGAAGGCCTTCCAAAAGGCTTATTATAACAGGTATGGAGGTG

GGGCCAATGAATTATCGGATGGGTACAACAAGGGAACTGGATTGGGTGCTGAGATCATTGGCACCT

TTGTTCTTGTCTACACTGTCTTCTCTGCCACTGATCCTAAGAGGAATGCAAGAGACTCCCATGTTCCTG

TATTGGCACCTCTCCCCATTGGATTTGCTGTGTTCATGGTTCACCTTGCCACTATTCCAATTACTGGCA

CTGGTATCAACCCTGCTAGGAGTTTTGGAGCTGCTGTTATTTACAACAAAGATAAGGCCTGGGATGA

TCAGTGGATCTTCTGGGTTGGACCTTTTATTGGAGCTGCCATTGCTGCTTTGTACCACCAATACATTCT

TAGAGCAGCAGCCATTAAGGCTCTAGGATCCTTCAGGAGCAATGCT**TAA**ATAGGCCTTTTAATTTTAT

TTGATAATTTGAAGAAAGAGGGTGGTGATGATGATGATGATGATGCTTAAGAAGAATAACAATGGA

TGAAGATGAGTGGTCAAGTTCTGTCTTTTAAGGGGTTCCTTCACTAATTTCTTTCTCAATCCCTTTCAA

GGATTTATAGGGAGAAATTTGGAGTGGAATTGTAAATACTAAGCAATATTTATGGTCAAGGGTGTCT

TTTCAATCCCTGTTGTTTTTGTTATGATTGTATTTTAAGTTATAATTATGTCACTTATGTCTATCCTTTGA

TCCTTATCTTATGATCTCCTCTTTTTTTTTTTTTAATCCATTATCTTCTGGGTGTATTTCTTCTTGTTTAAG

CATAATCAATCTAGTAAAATGGGTTGTCTTCG

>HbPIP2;5 Unigene3569

GCCTTGTACTGAAAAAAATTACATTAGATGCATTTTAGAAGAGCCGTTGCAGCTAATGGTGGGGTCA
AGCAAGCGAAGGAGGGAAAAAAATGAAGAAAAAGATGGTATTTGGTCGGCTAATTAAAATTTAAGT
TAATTATGTCATCAAACGATGAATATTATACTCTCTTAATCATATACCGTCCATCCTCTCTTCCATAAAT
ACCCCCTCTTTGCTCGCCTCTCTAACCACATATTCTCCACCCCCTTTCAGAGCTCACTAGCCACCAGAG
AAGCGCAAAATCAAGAAAATATATAGATCAATAATTCTCTC**ATG**GCTAAGGACGTGGAAGTTGCAGA
GAATCCTGGAGAATTCTCGGCCAAAGACTACCATGACCCTCCACCTGCACCTTTGATCGATGTAGAG
GAACTTGGAAAATGGTCCTTCTACAGGGCTCTTATTGCCGAGTTCATTGCTACCCTTCTTTTCCTTTAT
ATCACTGTGTTGACAGTAATTGGATATAAGAGTCAGACTGACCCTGCCAAGAATTCTGATGCTTGTG
GTGGTGTTGGTATTCTTGGCATTGCATGGGCCTTTGGTGGCATGATCTTTATTCTTGTTTACTGCACTG
CTGGTATCTCTGGAGGACATATTAACCCAGCTGTGACCTTCGGGCTGTTCCTGGGACGCAAGGTGTC
ACTAATCAGGGCCCTTTTGTACATGGTAGCACAGTGCCTGGGTGCAATATGTGGGTGTGGTTTGGTC
AAGGCCTTCCAAAAGGCTTATTACACCAGGTATGGAGGTGGGGCCAACGAACTCTCTTCTGGGTACA
GCAAGGGCACTGGTTTGGGTGCTGAGATCATTGGTACCTTTGTTCTTGTCTATACTGTCTTCTCTGCC
ACTGATCCTAAGAGGAATGCAAGAGATTCCCATGTTCCTGTATTGGCACCTCTTCCCATTGGATTTGC
TGTGTTCATGGTTCACCTTGCCACTATTCCAATTACTGGCACTGGCATCAACCCTGCTAGGAGTTTTG
GAGCTGCTGTTATCTACAACAAAGACAAGGCCTGGGATGATCAGTGGATCTTCTGGGTTGGACCTTT
TATTGGAGCCGCTATTGCTGCTTTCTACCACCAATACATTCTGAGAGCAGCAGCCATTAAGGCTCTAG
GATCCTTCAGGAGCAATGCT**TAA**TTTAGTTTTTAATTTATTTGATAATTTGAAGAAAGATGGTGATGA
TGCTTGAGAAGAATAACAATGGATGATGATGAATGAGCAAGTTGTCTTTTAAGAAGGGGTCCCTTCC
CCATTTTCTCTCTTTTCCCTTTCAAGGGTTTTGAGGGAGAAAATTGGAAAAGCCCATGGAATTGTAAA
TAATAAAAATTTATGGTCAAGGTGGGGTGTCTTTTCTATCCCTGTTGCTTTTGTTATGATTTGTATTTTT
AAGTTGTCACTTTTGTTTATCCTTTGATCCTTATCTTATGATCTTCAGCTCCTAGTTTTTCCCAATCCATT
ATCTTTTGGGTGTATTTCATCTTGATTGTGCTTAATTAATCTAGTAAAATGGGTTTTGTAGTCTCTCTTT
TTCTTTATCAT

>HbPIP2;7 Unigene4501

ATAATTTATCAGCTTTCCAAAAGCAAAGAGAAAAAAGCCAGAAATAACCGTTGTAGCTTGTATGCAT
TTTGGCTTGGATGCGTGGCTGACGCATCCCTGCTCTCTTACATTAATTTGATGATTACAATCACATGGT
TATCTTTTCATTTCTTCCCAATATACCCTTATCTTATCCATTAAACCCTTCCTTCTCTCGTTCTTCTTTAAA
AGCGACCCATTCTCCTCACTCCTCCATTACTCACTTACAAGACAACCTTCAATCTCACAAGACACTGCT
TTTGGTTTTGGGTTTGACAGAGATCAAAAGAGGAGAGAGAGAGAGAGAGAGAGAGAGAGAGAGA
AG**ATG**GCTAAGGAAGTGAGTGAAGAAAGGCAGCCAGGTAAGGATTATGTTGATCCACCACCAGCTC
CTCTAATCGACATGGCTGAGATCAAGCTCTGGTCTTTCTACCGTGCTCTTATAGCTGAGTTCATAGCC
ACTCTTCTTTTCCTCTATATCACTGTAGCTACTGTAATTGGCTACAAGAAGCAAGCTGACCCTTGTGCT
GGAGTTGGCCTTCTGGGTATTGCATGGGCCTTTGGTGGCATGATTTTCATTCTGGTTTACTGCACTGC
TGGTATCTCCGGTGGTCATATTAACCCGGCGGTCAGTTTTGGACTGTTCTTGGCGAGGAAGGTTTCA
CTAATCAGGGCTGTGGCTTACATGGTGGCTCAGTGCTTGGGTGCAATTTGTGGTGTTGGGTTGGTGA
AAGCATTTATGAAGCATCCATATAATGGCCTCGGAGGTGGTGCAAACACAGTGGCACCTGGCTACAA
CAAAGGCACTGCTTTGGGTGCTGAGATCATCGGCACTTTTGTACTTGTCTACACTGTTTTCTCTGCAAC
TGACCCTAAGAGGAGTGCACGTGACTCTCACGTCCCTGTGTTGGCTCCTCTTCCAATTGGGTTTGCTG
TGTTCATGGTCCACTTGGCAACAATCCCCATCACTGGTACTGGTATTAATCCTGCTAGGAGCTTTGGT
GCTGCAGTTATCTACAACAATGACAAAGCCTGGGATGATCATTGGATTTTCTGGGTTGGTCCTTTCGT
TGGAGCACTTGCAGCAGCAGCATACCATCAATACATACTGAGAGCAGCAGCCATCAAAGCTTTGGGA
TCTTTCCGCAGCAACCCCACCAAC**TAA**AGAAAGAGAAATCTGCACAAAAAAAAAAGAAAAAAAAAA
GCCTCTTTCTCTCTACTCTTCTTTTTCTCTTTCAAATTGTTTGTTTGTTTGTGTGTATTATGAGAGGATTA
TGATGATAACCCTTTTTCTTTTTCACTTCAATCTTTAATCTTTCTTTGTTAATTTAATTAGTCTCTGGCTC
TGTATCATTGTTATCAAGTAGTGCTGTTTTTCTTTTTCCTCTTTTCACACATTGCCCTCTGCTGCAGTGA
AAAGATTCCTCTCTATCCTTCCACAAAAATCTTAACTAATTTTGATTAATTTGCCTGTAAATGACAAAA
AGAAAAAGTACCCTTAACCAAATTAGCTTAAATTTAATTATAAGTCTGATTTATTCTG

**File B.** Phylogenetic analysis of identified HbPIPs with the complete set of *Arabidopsis* PIPs encoded in the genome. The tree was constructed using the pairwise distance and Neighbor-Joining algorithm with MEGA 4.0 software (Tamura et al. 2007) and bootstrap value was set to 1000. The distance scale denotes the number of amino acid substitutions per site.

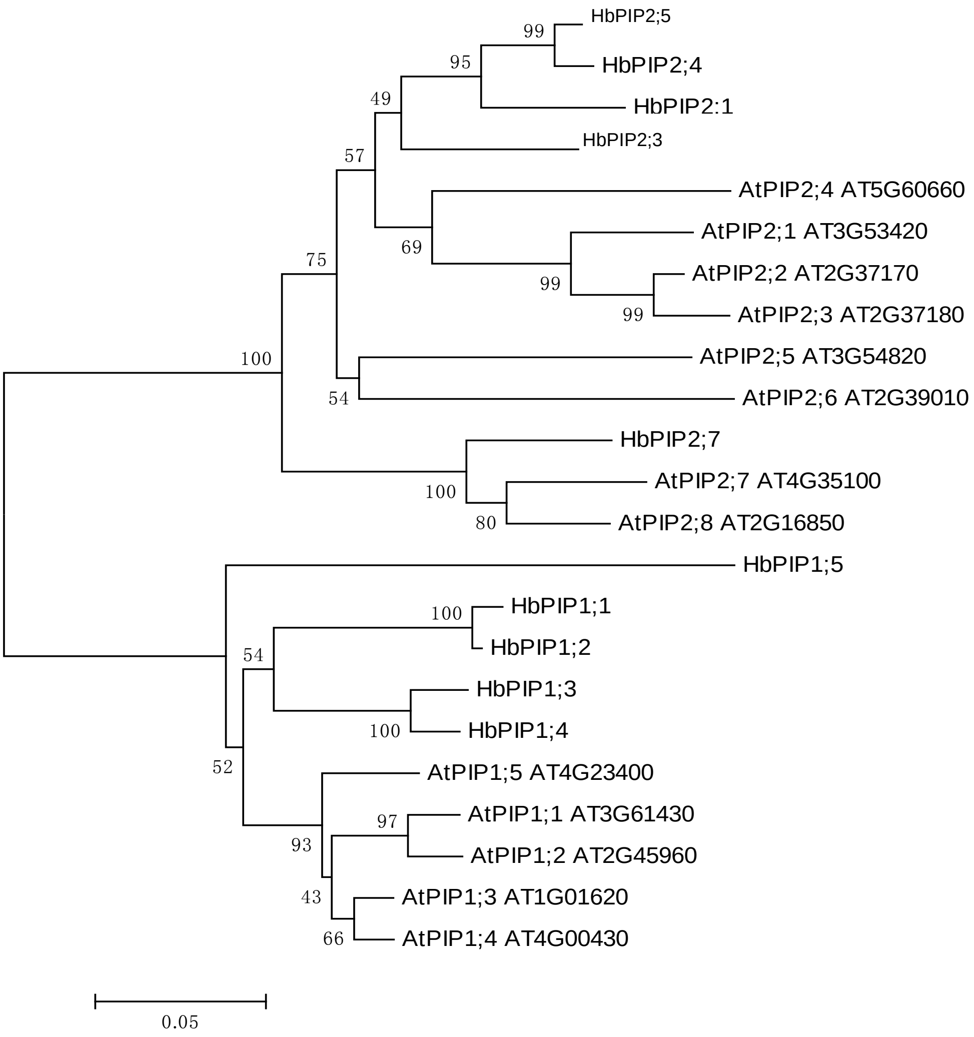

Supplement: S1 File — File A, The nucleotide sequences of ten transcripts identified in the rubber tree latex transcriptome. File B, Phylogenetic analysis of identified HbPIPs with the complete set of Arabidopsis PIPs encoded in the genome. (PDF) [file pone.0125595.s001.pdf]
