# Supplementary material for: Regulation of HbPIP2;3, a Latex-Abundant Water Transporter, Is Associated with Latex Dilution and Yield in the Rubber Tree (Hevea brasiliensis Muell. Arg.)
Source: PLoS One. 2015 Apr 30;10(4):e0125595. doi: 10.1371/journal.pone.0125595 (PMC4416032; doi:10.1371/journal.pone.0125595)

S2 File. The nucleotide sequence and gene structure of *HbPIP2;3*

File A. The transcriptional region of *HbPIP2;3* gene and its deduced coding protein. The gene structure was defined with Illumina reads; The coding region is marked with uppercase letters, under which is its deduced animo acids; The transcribed untranslated regions are marked with lowercase letters, including three introns, 5’ UTR and 3’ UTR; The primers used for PCR amplification are labeled with single underline; SNPs between the cloned ORF and the genome sequence are shown in bold letters.

```
   1 aaaaattgtttctcaatattaaatggaaaaatgactaaataagtactttatgaaataaaa
  61 aataaaatccttggaaacaaagaaaatgaaaaaaaaaagaaaaaaaaaatgaaaaaaaaa
 121 cagcctttattttacccgctctccttcaccatttatacactcgctcagctcagcttctct
 181 gtatccaaagtctcaaaaccaaaacgccaacccccctctctctccctctctatccccttat
 241 atacaaaatcttgctgtttctctttgctgagggtcgaccATGGTGAAGGACGTGACAGAA
   1                                        M  V  K  D  V  T  E
 301 CAAGGATCATTCCCAGCGAAGGACTACCATGACCCACCACCAGCACCATTGATTGATGCG
   8 Q  G  S  F  P  A  K  D  Y  H  D  P  P  P  A  P  L  I  D  A
 361 GTGGAGCTAACCAAGTGGTCATTTTACAGGGCCTTGATTGCTGAATTTATAGCAACTTTG
  28 V  E  L  T  K  W  S  F  Y  R  A  L  I  A  E  F  I  A  T  L
 421 CTCTTTCTTTACATCACTGTTTTGACTGTGATTGGATACAAAAGCCAGACTGATCCTGCA
  48 L  F  L  Y  I  T  V  L  T  V  I  G  Y  K  S  Q  T  D  P  A
 481 AAGAATGCTGACTCTTGTGGTGGTGTTGGAATTCTTGGCATCGCTTGGGCCTTTGGTGGC
  68 K  N  A  D  S  C  G  G  V  G  I  L  G  I  A  W  A  F  G  G
 541 ATGATCTTTATTCTTGTTTACTGCACTGCTGGTATTTCAGgtgagatatatagctttaat
  88 M  I  F  I  L  V  Y  C  T  A  G  I  S  G
 601 tttcctcctctttccttctttcttccttccctagaacagttttcagagcttcaagatttc
 661 tttttgtagcttcttcgaatggtaaatgtttttttacagtagtttttccattattttccca
 721 cttcttcaccaaggagtaagtattctttgctcatcaagttttcttgggaagcaaacagat
 781 tattagtttctttgttaattgcgtactatctttaatgaatattatgttcctttttcttaa
 841 gctagatacgaaaaaggatttgccttttatacttgaaaaagaaaaaaaaagaaaagaaa
 901 agcattttcctttttaaagatttctcaagaaaagtttttgtttttagaaatcagattttc
 961 ttaagaaaagttgatgagcttgtacccagacattaaacacaggcacagccttggcttttt
1021 cttcaattgttagcgtattttagcagatagttacgtgatttttcaaatctgcgcagGAGG
 102                                                          G
1081 GCACATTAACCCAGCAGTGACATTCGGGTTATTCCTGGCCCGAAAGGTCTCACTGGTACG
 103  H  I  N  P  A  V  T  F  G  L  F  L  A  R  K  V  S  L  V  R
1141 GGCCGTCATGTACATGGTGGCCCAGTGCTTGGGAGCCATAGCCGGTGTCGGATTGGTGAA
 123  A  V  M  Y  M  V  A  Q  C  L  G  A  I  A  G  V  G  L  V  K
```

```
1201 GGCCTTCCAGAGTTCCTTCTATAAGAGGTATGGTGGTGGGGCCAACAGTCTGGCTGCTGG
 143  A  F  Q  S  S  F  Y  K  R  Y  G  G  G  A  N  S  L  A  A  G
1261 GTACAGCAAAGGCGTTGGATTGGGTGCCGAGATCATCGGGACTTTTGTTTTGGTCTACAC
 163  Y  S  K  G  V  G  L  G  A  E  I  I  G  T  F  V  L  V  Y  T
1321 AGTGTTTTCCGCCACAGATCCGAAGAGGAATGCCAGAGACTCCCATGTGCCGgtatgtat
 183  V  F  S  A  T  D  P  K  R  N  A  R  D  S  H  V  P
1381 cataacatttctcatagaaattaattctctgagacttttgggctataattagtaatcact
1441 aattaattgttctcataaagatttgcatgcttgtttcacatgtgctatggtatgatatca
1501 tgaaccatgtgttttgctgactttagcaaatcttaatgcctgtttatatattaatgatta
1561 acaatttttttttatttaattgtaaaattagGTTTTGGCTCCACTCCCAATTGGATTTG
 200                                 V  L  A  P  L  P  I  G  F  A
1621 CTGTATTCATGGTTCACTTGGCCACCATTCCAATCACTGGAACCGGCATCAACCCAGCCA
 210   V  F  M  V  H  L  A  T  I  P  I  T  G  T  G  I  N  P  A  R
1681 GGAGTCTAGGAGCTGCTGTTATCTACAATCAGGACAAGCCCTGGGATGATCATgtcagta
 230   S  L  G  A  A  V  I  Y  N  Q  D  K  P  W  D  D  H
1741 tatataaacatattcatcaacttaattatggttattaattaaacatggtaatgggtactt
1801 aattttcttgttaaattatttttttcttgcagTGGATCTTTTGGGTTGGACCCTTCATTG
                                                     T
 247                                 W  I  F  W  V  G  P  F  I  G
1861 GTGCAGCCATTGCAGCCTTCTATCACCAATTCATCTTGAGGGCAGGAGCTGTGAAGGCTC
 257   A  A  I  A  A  F  Y  H  Q  F  I  L  R  A  G  A  V  K  A  L
1921 TTGGATCATTCAGGAGCAACCCAACTGTTTAAgggaagaaataatttttctaaactaata
         G
 277   G  S  F  R  S  N  P  T  V  *
1981 atgaaggaaaagcatgtgctggttggtttcattattagcccttctggtgtgttctttgtt
2041 gttgggggggttgaaaatgtgagaagagaggtttgaaagaattatggaatttgtagatat
2101 aaaagcctctttgaaaatgggaggcttttggttatcccttttttatttgttttgtttgat
2161 ttcacttagcaatgttatgatggttttcttggttggttcatcatcatcatctattgtcta
2221 tttttcttctttgcacttattatgcttgtaagaaaagttaggctcttttgcttttatta
2281 taagtgtaggctatgcttgttttattatgagctttaagtttcattttcacatattatgtt
2341 tccatatttttttccaaatcaagtattcatttcataa
```

File B. The schematic gene structure of *HbPIP2;3*. Exons are shown as blue boxes, while UTRs and introns are shown as black lines.

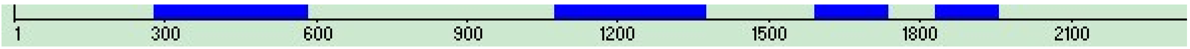

Supplement: S2 File — File A, The transcriptional region of HbPIP2;3 gene and its deduced coding protein. File B, The schematic gene structure of HbPIP2;3. (PDF) [file pone.0125595.s002.pdf]
