# Supplementary material for: Regulation of HbPIP2;3, a Latex-Abundant Water Transporter, Is Associated with Latex Dilution and Yield in the Rubber Tree (Hevea brasiliensis Muell. Arg.)
Source: PLoS One. 2015 Apr 30;10(4):e0125595. doi: 10.1371/journal.pone.0125595 (PMC4416032; doi:10.1371/journal.pone.0125595)
Supplement: S1 Table — (DOC) [file pone.0125595.s003.doc]

**S1 Table.** Gene specific primers used for quantitative RT-PCR assay of aquaporin expressions in rubber tree latex

| *HbYLS8* | F: 5’- GGGCTCTCAAGGACAAGCAA-3’ |
| --- | --- |
|  | R: 5’-GGAGCAATAACCAAACCACGA-3’ |
| *HbPIP2;1* | F: 5'-TGCCGCAATTGCTGCATTCT-3' |
|  | R: 5'-AAAACCATGAACCCAAGCAC-3' |
| *HbPIP2;3* | F: 5'-CGTTGGATTGGGTGCCGAGAT-3' |
|  | R: 5'-CCAGGGCTTGTCCTGATTGTA-3' |
| *HbPIP2;7* | F: 5’- CATATTAACCCGGCGGTCAG-3’ |
|  | R: 5’-CACCAACCCAACACCACAAA-3’ |
